# Reading in the city: mobile eye-tracking and evaluation of text in an everyday setting

**DOI:** 10.3389/fpsyg.2023.1205913

**Published:** 2023-10-18

**Authors:** Kirren Chana, Jan Mikuni, Alina Schnebel, Helmut Leder

**Affiliations:** ^1^Department of Cognition, Emotion, and Methods in Psychology, Faculty of Psychology, University of Vienna, Vienna, Austria; ^2^Department of Foreign Languages and Literatures, University of Verona, Verona, Italy; ^3^Vienna Cognitive Science Hub, University of Vienna, Vienna, Austria

**Keywords:** eye movements, mobile eye tracker, reading, signs, urban street environment, aesthetic evaluation

## Abstract

Reading is often regarded as a mundane aspect of everyday life. However, little is known about the natural reading experiences in daily activities. To fill this gap, this study presents two field studies (*N* = 39 and 26, respectively), where we describe how people explore visual environments and divide their attention toward text elements in highly ecological settings, i.e., urban street environments, using mobile eye-tracking glasses. Further, the attention toward the text elements (i.e., shop signs) as well as their memorability, measured *via* follow-up recognition test, were analysed in relation to their aesthetic quality, which is assumed to be key for attracting visual attention and memorability. Our results revealed that, within these urban streets, text elements were looked at most, and looking behaviour was strongly directed, especially toward shop signs, across both street contexts; however, aesthetic values were not correlated either with the most looked at signs or the viewing time for the signs. Aesthetic ratings did however have an effect on memorability, with signs rated higher being better recognised. The results will be discussed in terms aesthetic reading experiences and implications for future field studies.

## 1. Introduction

In everyday life, perception is a powerful tool that guides us through our complex environments and, by controlling our attention, directs us toward relevant, interesting events and objects (Bruce et al., 2003; Clarke et al., 2013). In a figurative sense, the way in which people engage with and assimilate their surroundings requires what can be regarded as ‘reading the city,’ highlighted in *The Image of the City* (Lynch, 1960) to denote how the urban environment is perceived (Silva Gouveia et al., 2009). This also includes the ability to locate and deduce meaning from visual elements in everyday settings (i.e., taking notice of directions or road signs while navigating toward a destination), i.e., ‘everyday reading.’ Reading thus extends beyond the literary context and instead could be considered more a cognitive sub-skill of visual exploration and understanding the environment.

In a narrower sense, and also as the essence of signs for reading, text in today’s urban environments is omnipresent - it is almost, if not, impossible to walk through a city without reading. Reading text can be regarded as one of the most outstanding features that make us human, and in most cases, when we are exposed to text, cannot be avoided (Jean, 1992; Robinson, 2009). From a literary perspective, reading involves decoding characters or symbols, identifying them as words, and subsequently interpreting their meaning (Findlay and Gilchrist, 2003). As such, it is clear that reading text is a key requisite in daily affairs, and our urban civilizations are inundated with written information, so much so that we are perhaps gripped by its consumption (Robinson, 2009).

Nevertheless, despite the importance and relevance of reading in our daily lives, the sheer number of reading experiences encountered by an individual on a daily basis in ecologically valid settings (e.g., street environment, etc.) is often overlooked, and little is known about how reading is meaningfully distributed in our lives. A possible reason could be the difficulties of performing such observational studies in highly ecologically valid settings, which requires many resources (e.g., devices to measure participants’ attention, inviting participants to a testing place with enough experimenters, etc.). However, although this is a challenging task, it is important to describe and understand how people actually divide their attention in a natural context, as the testing contexts here (e.g., well-controlled laboratory, complex urban street environments) might play a crucial role.

In the current paper, through two field studies, we present a first step to bridge this gap. Specifically, we investigated *how* people divide their attention to readable objects of any kind, while they freely walked around an urban street environment, and specific reading occurrences (i.e., toward shop signs), using mobile eye tracking devices to record eye movement patterns of participants. Furthermore, we also aim to investigate *what* is important for text to capture one’s attention. More specifically, we focus on how aesthetic values in texts in everyday life affect our perception in terms of attention measured *via* viewing time (e.g., do more beautiful signs receive longer looks than less beautiful ones?) as well as its memorability (e.g., do people remember more beautiful signs better than less beautiful ones?).

In the following section, we first provide a general overview of how reading/reading behaviour in everyday life has been studied in psychology and in other fields. Secondly, we argue how visual aesthetic qualities can be related to and impact reading. Finally, we present two field studies in which we assess the relationships between real-world scene-perception, reading behaviour, aesthetic appreciation, and memory.

### 1.1. Studies of everyday reading in the field of psychology

The decision of where to look is imperative in the undertaking of many everyday activities. With an average of three fixations made per second (Cristino and Baddeley, 2009), this is a task we must do incessantly. Naturally, our gaze is guided by our surroundings and potential obstacles when walking, in relation to the principal goals of locomotion; i.e., to walk safely from one location to another (Warren, 1998). However, when people are able to walk freely in a space with no obstructions, there is a possibility for gaze to fall upon other items that are present within the peripheral field. In the context of urban settings, this can be guided by the intention of walking along a city street (e.g., leisure, shopping, orientation), or even a lack of purpose. The latter is apparent for the flâneur, a person who wanders aimlessly, ‘mapping their surroundings with their feet’ as a means of creating a ‘meaningful experience with the city’ (Elkin, 2017). At first, it seems such an act would result in one becoming lost in a metropolis, but in fact Lynch (1960) noted it is a rarity to be completely disoriented within built environments. This is arguably since legibility and wayfinding are important features in city design, to the extent that we are bombarded with a myriad of wayfinding devices. These are information systems with textual and graphic sign elements that contribute to our identity with (sense of belonging), understanding, and experiencing of the city (Lynch, 1960; Bauer and Mayer, 2008; Huerta, 2010; Harland, 2015; Clark et al., 2017).

In fact, there have been a few studies where researchers examined how people divide attention toward texts compared to the other components. For example, research in scene viewing has demonstrated that text generally does draw visual attention. Cerf et al. (2009) found, relative to objects of a comparable size and location, text and faces attracted more attention. Wang and Pomplun (2012) conducted a series of laboratory experiments to consider the attraction to text in images of real-world scenes. They found text drew more attention than non-text objects and control regions. Furthermore, in the field of marketing, print advertisements also highlight brand, pictorial and text elements as key to capturing attention, with text arguably being the most central (Ogilvy, 1985; Belch and Belch, 2001; Pieters and Wedel, 2004).

It is worth noting, however, that such laboratory-based studies lack ecological validity. Indeed, laboratory experiments are important to assess the impacts of target factors while controlling confounding factors. However, to understand how people are dividing their attention to objects in urban scenes, the ecological validity in the testing setting seems to be crucial. In a laboratory setting, one might have looked at still images containing a limited number of items while seated. Yet, the actual interaction with visual stimuli in natural (i.e., real life) urban scenes is obviously far more complex. In a natural urban scene, there are not only multiple forms of text present, but also the text contends with a multitude of objects for attention, while the observers are moving around with more physical freedom and flexibility - a setting very different compared to that of a well-controlled laboratory study. Further, as aforementioned, while study participants tend to engage in specific tasks in a laboratory, actual pedestrians can have very different purposes for engagement in urban settings, which can also impact where, what, and how to search the environment (Droll and Eckstein, 2009). As such, the results delivered from oversimplified situations in a laboratory setting probably do not reflect the actual behavioural patterns in a real-life setting.

Hence, although past literature demonstrates the potential of text to capture our visual attention more than other components, in order to describe how the text attracts our attention in a real urban environment, it is quite important to observe natural behaviour in a highly ecologically valid setting, which has been rarely assessed in the past literature. In the present paper, we tackle this limitation by conducting field studies in real urban settings, using mobile eye-trackers to describe participants’ visual attention, and assess how people divide their attention toward text elements in a real urban environment amongst the other visual elements.

### 1.2. Aesthetic viewing in everyday settings

The matter of *what* determines the attentional guidance to text remains a topic up for debate. In relation to attentional processing, image-related features that are highly salient (i.e., colors, contrast, etc.) capture attention in a bottom-up fashion (Janiszewski, 1998; Itti and Koch, 2001). For example, the text element’s relative size is a possible saliency feature that captures attention in this way (Janiszewski, 1998; Pieters and Wedel, 2004). However, such studies do not provide a complete overview and often ignore some features from the reading literature. One candidate not addressed so far is the aesthetic quality of the text elements. Like any other designed visual object, text elements can elicit different aesthetic experiences, such as beauty.

Aesthetics^1^ have been shown to influence how we direct our attention in our environment, with aesthetic liking eliciting longer viewing time (Shimojo et al., 2003; Holmes and Zanker, 2012). On this basis, our sense of beauty guides what we experience by determining where we tend to look – and what binds our looks. This is in line with research which argues that visual attention is not necessarily directed to low-level features,^2^ but toward task-related areas in a scene that provides the greatest meaning or informational value (Itti, 2005; Torralba et al., 2006; Einhäuser et al., 2008; Kollmorgen et al., 2010; Pilarczyk and Kuniecki, 2014; Wu et al., 2014; Henderson and Hayes, 2018; Peacock et al., 2020). From this perspective, considering that the aesthetic experience provokes complex psychological processes, such as meaning making and memory integration (Leder et al., 2004; Pelowski et al., 2016 for review), it seems theoretically plausible to postulate that texts, which are high in aesthetic experience, might also capture our attention more.

Similar findings are shown in other domains of research. For example, preferred faces and artworks have been shown to bind visual attention (Shimojo et al., 2003; Leder et al., 2016). Such studies have implemented a free-viewing paradigm, which involves looking at stimuli without a specific task, and have shown that aesthetically preferred objects (as indicated in follow-up evaluations) bind visual attention. Using this design, images were presented of natural everyday scenes that depicted two people, with the more attractive faces evoking longer looks (Leder et al., 2010, 2016). Similar effects had also been shown for artworks (Goller et al., 2019; Mikuni et al., 2022), or more beautiful images (Mitrovic et al., 2020) of the visual aesthetic sensitivity test.

Interestingly, the attention toward stimuli that are high in aesthetic experience has been demonstrated not only in the laboratory but also in a field study with a more comparable situation to that of our study. Mitschke et al. (2017) examined viewing behaviour, in an outdoor natural environment, during a free exploration walk along a canal pathway containing art/aesthetic objects, e.g., graffiti or sculptures. Similar to the findings from laboratory studies, individual aesthetic evaluations (i.e., liking, interest) of objects within the natural setting were positively correlated with the total fixation durations. Thus, even in such an everyday environment, in which there are many visual stimuli, and subsequently great individuality/variability in viewing behaviours, the beauty of objects played an important role in how people direct their looks.

In this light, perhaps also viewing behaviour in everyday reading could be related to aesthetic quality/preference. Accordingly, in the present study, we assess how subjective aesthetic quality of the text elements and viewing behaviour (i.e., amount of divided attention, viewing time) are related.

### 1.3. Real world memory and visual attention

An essential purpose of text is the preservation of information (Jean, 1992; Jaderberg et al., 2016), therefore it is not only what text people look at in natural scenes that is of interest, but also whether they remember it. Despite its fundamental functions in urban environments, research exploring memory and vision is rarely carried out in environments with greater visual complexity. This is despite the intuitive suggestion that more natural stimuli will have a stronger memory trace when viewed for greater lengths of time (Irwin and Zelinsky, 2002; Melcher, 2006). As such, the question of what enhances the memorability of the texts remains open and warrants further investigation.

Past studies have suggested attending to elements of a scene is essential for the encoding of a representation (Hollingworth and Henderson, 2002), meaning it is possible that longer looks give rise to improved memory. Following our arguments from the previous section, it seems plausible to postulate another relationship between aesthetic quality and texts elements; that is, if longer looking times are found for more aesthetic and more beautiful objects, and longer looks enhance the memorability of the items, then higher aesthetics might result in higher memorability (e.g., when one finds a text element more beautiful, it would be remembered better than less beautiful ones). Accordingly, we also assess how the subjective aesthetic quality of the text element and its memorability are related in the present study as well.

### 1.4. Present study aims and hypotheses

The current paper focuses on the engagement with reading the environment in a naturally-occurring urban street setting. We especially aim to study viewing behaviour toward text in an everyday setting, given that previous studies (in labs/real-world scenes) suggest text inevitably captures attention, yet were lacking ecological validity. Owing to the situation of carrying out studies in natural outdoor environments, it was not feasible to actively control/manipulate the testing environment in the current studies. As such, an exploratory approach was undertaken with broader objectives in order to describe natural reading behaviour in an ecologically valid setting and its relation to aesthetic value.

In the present paper, we set three distinct research questions: (1) What is the prevalence of reading? (2) How does viewing behaviour correlate with the aesthetic qualities of text? (3) What are the effects of text differing in aesthetic quality on memory? We addressed these questions in the two studies with the following hypotheses corresponding to each research question: (1) As shown in the past literature, in real-world walks in urban environments, more fixations would fall toward signs and text compared to the other visual objects in the testing environment; (2) In evaluating the major visual elements for aesthetic preferences, more aesthetically valued texts also yield longer fixation times; (3) Based on the reported relationship between aesthetic experience and viewing time as well as between memorability and viewing time in the past literature, we hypothesise that more aesthetically valued texts are better recognised.

To address our three research questions and test our three hypotheses, two field studies in different urban street environments were conducted. Study 1 considered viewing behaviour of text across a section of the Mariahilferstraße shopping street in the 6th district of Vienna. This section forms the beginning of a pedestrianised shopping area which is popular amongst tourists and locals. Mariahilferstraße is a very busy shopping street, with a greater number of shops, advertisements, and buildings with busy traffic. The street is also lined with trees and largely pedestrianised, therefore there is presumably a higher propensity to view nature and people. It can therefore be considered as an everyday urban environment, which can be found in many different cities and countries, and in which various forms of text, as well as a variety of other objects, have the potential to capture one’s attention. Such testing environment was especially suitable to test our first research question: What is the prevalence of reading *amongst other available objects*? The first study also served as a proof of concept, that our methods can reasonably be employed to test our hypotheses. Study 2 also considered the viewing behaviour of text across a section of the Siebensterngasse, wherein the circumstances were thought to be quite different than in Study 1. The second street represented a supposedly more mundane street, in that it is not pedestrianised and comprises a selection of shops, cafes, and public transport representative of the average Viennese street (see Furchtlehner and Lička, 2019; Furchtlehner et al., 2022), and thus also a higher propensity to view traffic and people as a reflection of the specific street (e.g., Geruschat et al., 2003). By conducting similarly structured field studies with two urban streets, which differ in terms of street characteristics and available objects, we aim to also test the generalizability of reading behaviour, and to report, if any, possible differences as a reflection of the specific street.

We note our focus in the present studies is on reading behaviour in the street environment in general (e.g., attention toward advertisement, traffic/shop signs etc.). However, especially to examine the relationship between viewing behaviour/memorability and aesthetic quality in texts, we explicitly focus on the shop signs. This decision was made, as in our testing streets, there are a variety of shop signs, which would result in a variety of aesthetic qualities in texts, while there seem to be less variety in other types of texts.

## 2. Study 1

### 2.1. Materials and methods

#### 2.1.1. Participants

A sample of 39 participants (26 female, 13 male; *M*_age_ = 24.25, *SD*_age_ = 3.60, range: 19–32 years) were recruited *via* the University of Vienna. The average height of the participants was 172.35 cm (*SD*_height_ = 8.66, range: 155–187 cm, two missing due to recording issues). One participant used a wheelchair, however could not be included in the eye-tracking analyses due to data loss (detailed further in Data Preparation section). All participants included in the analysis had normal or corrected vision and were native German speakers. Anonymity was guaranteed with the use of personal identifiers, and participants received monetary compensation (10 €). The study corresponds with the ethical standards of the Declaration of Helsinki and the ethical regulations at University of Vienna. For more detail regarding participant information refer to OSF.^3^

#### 2.1.2. Apparatus

The Pupil Labs wearable eye-tracking headset (Pupil Core) was utilised to track the eye movement patterns of the participants during their walk. The portable glasses are equipped with three adjustable cameras to record each of the participants’ pupils (Eye Camera, 200 Hz, 192x192px) and the outer environment (World Camera, 30 Hz), to monitor where individuals are looking. The eye-tracking data was collected on a Windows Microsoft Surface Pro 4 tablet, running the software EyeRecToo (see Santini et al., 2017a; Reitstätter et al., 2020). Calibration was administered with a single ArUCo marker with a spiral movement pattern, as suggested in CalibMe (Santini et al., 2017b). This uses numerous calibration points, with the spiral movement allowing the wearer to change the spatial distance and head angle for better gaze estimation within a mobile setting.

#### 2.1.3. Setting

**Figure 1:**
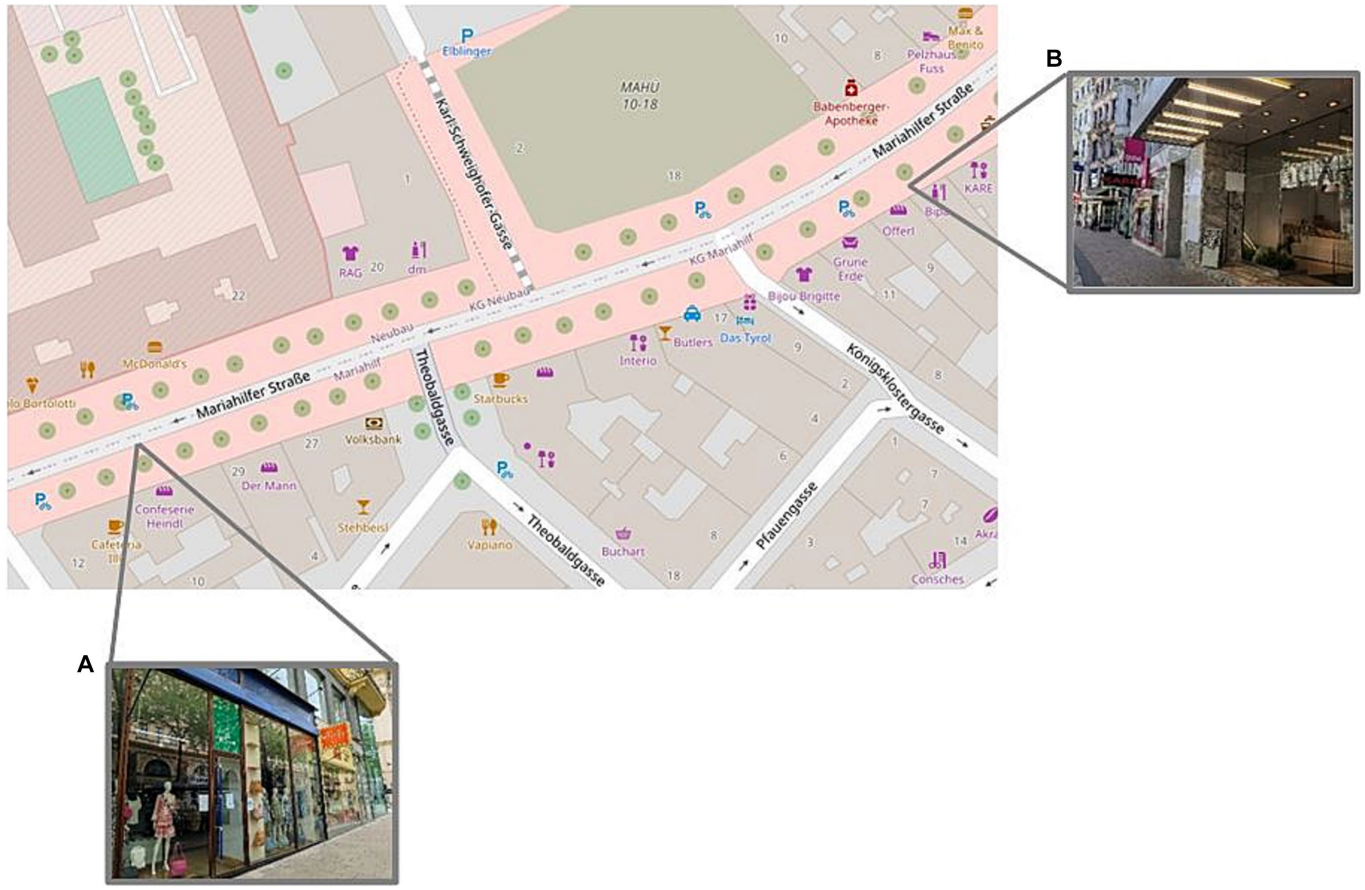
OpenStreetMap image, depicting the eye-tracking route along Mariahilferstraße 31 to 7 in Vienna’s sixth district. The images denote the start **(A)** and finish **(B)** positions along the eye-tracking path.

A 250 m section of Mariahilferstraße was chosen for the eye-tracking path. The start point was set at Mariahilferstraße 31 (48.2004918,16.3573532; 6,924 + 6X Vienna) and the endpoint was set at Mariahilferstraße 7 (48.20118,16.359881; 6,926 + C2 Vienna), where the ‘BIPA’ drugstore is located, which marks the point at which the street begins to curve so that further road traffic and more distant landmarks at Museumsquartier can be seen (see Figure 1). Further, although this part of Mariahilferstraße is not completely pedestrianised, only one small side street (Königsklostergasse) without any specific road markings fell within this 250 m path, which meant there would be minimal traffic interfering with the eye-tracking path. Given this portion of the street is on a slope, participants walked both uphill and downhill, as it was not apparent which direction was best or would elicit better/different viewing behaviour.

All recordings took place when the street was less busy and the majority of stores were still closed. It is, however, noted there was extensive construction work on the opposite side of the street, which progressively moved further within the testing section of interest and could potentially have caused a marked difference in viewing behaviour. Though, given this was a field study and external variables cannot be controlled, this was accepted as another distractor as there were many other facets that meant there was variation in the street conditions for each participant (i.e., different people, new graffiti).

#### 2.1.4. Eye-tracking procedure

Testing took place in 2 week blocks across two waves, in June and September 2021. Eye-tracking data was collected across all days during this period (Monday to Sunday, between 8 am-10 am) to cover any notable differences to the environment across the span of a week. The general street setting was the same during this timeframe, but four particularly noticeable changes within the environment occurred: (1) escalation of construction work, (2) one store that had been vacated, (3) billboard advertisements that had been replaced, (4) prominent graffiti across the external wall of the Starbucks store (see Supplementary Table S1 for detailed descriptions). Participants were tested in two blocks but testing was stopped abruptly after 39 participants due to extensive construction work, which meant further testing was not feasible.

Upon arrival at the testing site, participants scanned a QR code with their mobile device to read the information sheet and electronically provide informed consent. Participants then provided an identifier and anonymized demographic information. After completing this, the eye-tracking glasses were set up and adjusted. Once the eye-tracking glasses were calibrated, participants were led to the starting point and given verbal instructions. Since this was a free-viewing paradigm, participants were not given a specific task and instead were instructed to walk along the path at their natural pace. This method was chosen since eye movements are influenced by the task at hand (Buswell, 1935; Land and Tatler, 2009). Therefore, without a specific task instruction, we expect viewing behaviour will be as natural as possible. They were informed they could move their head freely without restrictions and to raise their hand should they require help/feel discomfort at any point during the walk. The instructions were chosen to be as unconstrained and natural as possible, but to still ensure all participants would walk along the same path. As such, participants were unaware of the true purpose of the study, i.e., reading/observation of text and test of memorability. Participants then walked along the path, and an experimenter followed behind at a distance (approx. 3 m). At the endpoint, the experimenter stopped the recording, asked if the participant had any questions, and, after a short break, proceeded with recording the participant as they walked in the opposite direction. The side of the pavement and direction of the eye-tracking path (from A to B, then B to A) remained the same across all participants. When the participants returned to the original starting point, the eye-tracking session was concluded.

Participants were then seated facing away from the recorded section of the street and a post-viewing questionnaire was administered *via* a QR code. Here participants provided responses to open recall questions regarding what they noticed, remembered most clearly, liked and disliked. They also reported on 5-point scales (1 = not at all, 5 = very much) how much they liked, how familiar they were with and how often they visited the section of the street. Finally, they were asked if they would visit the section again in their free time and how comfortable they found the mobile eye-tracking glasses. Participants were then reminded they would be sent a further questionnaire to complete online *via* email within a 1–2 weeks timeframe.

#### 2.1.5. Follow-up session procedure

All participants were sent a further questionnaire to complete online 1–2 weeks after the initial in-person eye-tracking session. Firstly, participants performed a recall task, where they provided open-ended responses for any shop signs, architecture, street signs, people, advertisements, nature and text elements they remembered seeing from the eye-tracking walk; i.e., ‘Do you remember any shop signs that you saw during the walk?’ (translated from German). We note that participants’ open responses to the memory recall questions are not analysed in this paper as this was included exploratively to inform further study. For more detail regarding open recall refer to OSF (see footnote 3).

Secondly, participants performed recognition and rating tasks for the 40 photographs provided. Twenty photographs were taken of text sign elements from the eye-tracking path, and additional twenty photographs containing text from other areas within the city streets, which were used as distractors in the memory task.^1^ All photos were taken in landscape format with a ratio of 19.5:9 on a mobile phone camera (Huawei Mate40 Pro). Photos portrayed text from shop fasciae/signs and were selected at random from a larger corpus of images photographed from both directions of one side of the street. Though restricted to the text elements were present within the testing environment, we chose to focus on text signs due to the high prevalence and variation of shop signage within the street environment. We also reported such variations in terms of physical features (e.g., size, height, position, number of characters) (see Supplementary Table S2). For the recognition task, participants were asked to indicate whether the photographs of text elements from street scenes presented were from the eye-tracking path (yes/no dichotomous response). Participants also rated all 40 photographs for subjective aesthetic judgement on 7-Point scales, which are commonly used in empirical aesthetic research (beauty, interest, meaningfulness, likeability, attractiveness, emotionality, familiarity). The commonly used scales in empirical aesthetic research were adapted in the current study, as those scales, measuring aesthetic experiences, were not used in the past related studies of reading, and hence, there are no specific established findings in this regard. As such, all common scales are adopted not to overlook any possible effects of aesthetics in texts on viewing behaviour/memorability.

Afterward, participants completed a language history questionnaire (LHQ v3.0; Li et al., 2020), to see if participants have similar levels of reading ability and more exploratively, provide insight about their language use. In the LHQ, they reported demographics about their native language(s) and languages they had learned, as well as ratings of their proficiency in terms of reading, writing, speaking and listening (i.e., 7-point scale; 1 = very poor, 7 = native-like). Upon completion, participants were debriefed, thanked for their participation. We note the LHQ was asked to the participants for more explorative purpose, i.e., to develop further study ideas for future studies, and to analyse this data in relation to viewing behaviour and aesthetic was beyond the scope of the present study. Hence, the results are not reported here.

#### 2.1.6. Data preparation

The data for participants with complete eye-tracking video material was included for this analysis. With this criterion, the final sample of 25 (16 female) were included in the eye-tracking results below, reduced from an initial collection of 39 participants. Given the technical challenges faced with automatic labelling the eye movement data for the field camera footage, like Reitstätter et al. (2020), a manual approach was adopted using the software Eye Movement Coder (version 2.4). In this manner, frame length was used as a proxy of viewing time. This required looking at each video, on a frame-by-frame basis (33 ms per frame), and marking the start and end times for viewing specific objects of interest, in accordance with predefined labels for broad categories, similar to Mitschke et al. (2017) (see Table 1), and the 20 specific sign labels from the eye-tracking path (see footnote 4). Although a more laborious task, this allows objects of interest to be coded when partially occluded (i.e., when a text sign overlaps with another). These annotations were labelled by two researchers individually. Two videos were doubly checked to ensure consistency in the labelling and a unanimous decision was made for any annotations points where uncertain. On this basis, we were able to determine viewing time for object categories and sign labels (measured *via* frame length) as well as the frequency of fixations (i.e., multiple looks). Following from the manual annotations and data extraction, the eye-tracking data was then analysed with R environment (Version 1.1.423. R Development Core Team) using dplyr package (Wickham et al., 2023). Note that all analyses reported below were performed in the same R environment.

**Table 1:** Predefined object categories of interest for eye-tracking coding.

Category	Subcategories
Advertisements	Billboards, Posters, Sale Signs, Special Offers
Animals	Dogs, Pigeons
Architecture	Balconies, Facades, Ornate details, Windows
Graphic elements	ATM sign, Logo, Symbols
Nature	Bushes, Grass, Potted Plants, Trees
Other text	Graffiti, Plaques, Stickers
People	Adults, Children
Shop signs	All shop signs (Fascia/Hanging Signs)
Street signs	Bus signs, Road signs, Traffic signs, Tram signs
Traffic	Bicycles, E-Scooters, Scooters, Trams, Vehicles

For Study 2, ‘Animals’ was included, and ‘People’ were sub-divided by body/face.

Importantly, across the two studies in this paper, we only used fixation data points that were longer than 200 ms (i.e., where one frame is 33 ms, we removed fixations with a frame length of less than 6). Although minimum fixation duration is often set much lower at 100 ms as threshold that distinguishes between saccades and fixations, we adopted this more stringent fixation criteria (Salvucci and Goldberg, 2000; Deane et al., 2023; see also Supplementary Table S3; Supplementary Figures S1, S2) given there appears to be no clear consensus in past literature (for more extensive overview, see Holmqvist et al., 2011; Bojko, 2013). Overall, 8,530 fixations were made in entire recording. The number of fixations made by individual participants ranged from 123 to 609. The total time of the annotated videos was 1 h 57 min 20 s. Moreover, the guidelines for fixation criteria are often set from stationary eye-tracking data, and there is much less in regard to mobile eye-tracking, especially in field environments (Kiefer et al., 2014). This results in, out of 8,530 fixations, 1,381 fixations, which were included in the main analysis below.

### 2.2. Results

All of the data reported in Study 1 and 2 is available on OSF (see footnote 3). Prior to any further analyses, we calculated the average scores for information regarding the street location (5-point scales; 1 = not at all, 5 = very much) for the 37 participants in the final sample. Note that data from two participants were removed due to recording issues. This included participants’ liking (*M* = 3.81, *SD* = 0.81), familiarity (*M* = 3.84, *SD* = 1.14) and frequency (*M* = 3.19, *SD* = 1.05) of visiting this section of the street. Further, we asked if participants would visit this part of the street again; No = 7, Yes = 30. Additionally, the scores for comfort of the glasses were assessed on a 5-point scale (1 = not at all, 5 = very comfortable) to evaluate how natural wearing the mobile eye-tracking glasses felt to the participants. The average score for this question was 4.24 (*SD* = 0.80), and thereby, for the interpretation of the results, it was assumed the participants’ viewing behaviour was not constrained by wearing eye-tracking glasses and could be included in the analysis.

#### 2.2.1. The number of fixations per object category

**Figure 2:**
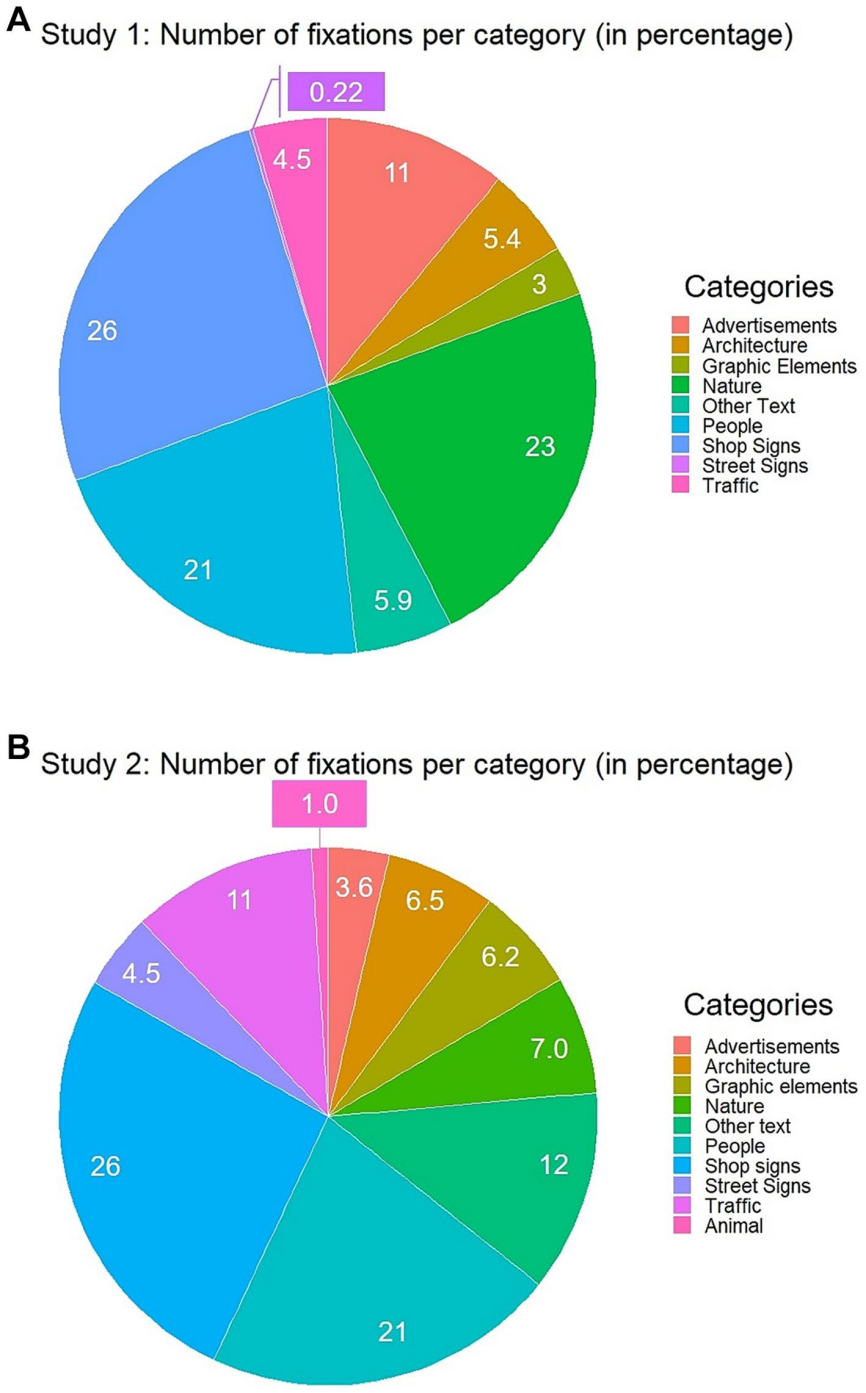
**(A)** Pie chart using all fixations made by all participants from the results of Study 1. **(B)** Pie chart using all fixations made by all participants from the results of Study 2. The number shown in the chart represents the percentage of fixations that fell into the given category.

Firstly, to test our first research question: ‘What is the prevalence of reading?’, we analysed which objects captured attention proportional to overall viewing time, and how many of these fixations fall toward specific signs and text more generally. To this aim, the total amount of fixations toward each category across all participants (%) were calculated (see Figure 2A). We note, since the results of the number fixations per object category should be compared visually between Study 1 and 2 to see the generalizability of our findings in different street environments, the results of Study 2 in this section are presented together with those of Study 1. As shown in Figure 2A, 70% of the total fixations fell into Shop Signs (26%), Nature (23%), and People (21%). The remaining 30% of total fixations fell toward Advertisements (11%), Other Text (5.9%), Architecture (5.4%), Traffic (4.5%), Graphic Elements (3%) and Street Signs (0.22%). Thus, our results show Shop signs are the most viewed component which people are paying attention to while walking street.

**Figure 3:**
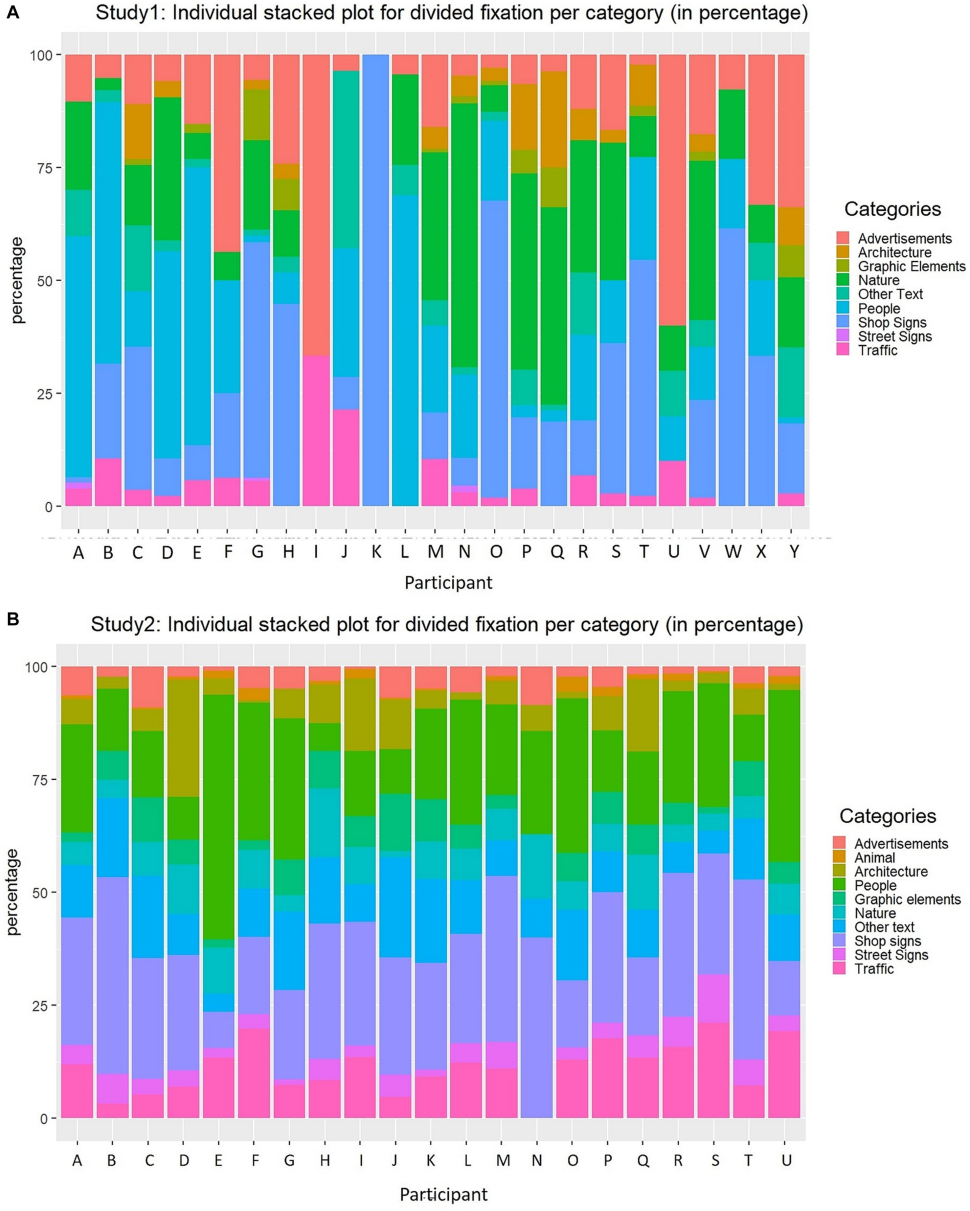
**(A)** Stacked barplot using fixations made by each individual on each category from the results of Study 1. **(B)** Stacked barplot using fixations made by each individual on each category from the results of Study 2.

Further, to see inter-individual differences, the percentage of the fixations on each category per participant was calculated (Figure 3A). The descriptive statistics for the percentage of the fixations are presented in the upper part of Table 2. The descriptive statistics were calculated as follows. First, the percentage of the fixation per category was calculated for each participant. These values per participant were then used to calculate the mean percentage of the fixation per category. In case one did not make any fixation on one object category, we used 0. Seeing Figure 3A; Table 2, not surprisingly, people divide their attention quite differently. For example, there were only three participants who made fixations on Street signs, while the other participants did not make any fixations at all. Similarly, almost only half of the participants made no fixations on Architecture and Graphic elements. This inter-individual difference can be also seen in large standard deviations (*SD*) for the mean percentage of those categories (Table 2). However, our results also show Nature, People, and Shop signs are the object categories to which the majority of the participants made fixations. Accordingly, despite huge inter-individual differences, there seems to also be general patterns in how people divide their attention in a street environment.

**Table 2:** Descriptive statistics for the number of fixations per category.

Category	Mean percentage	*SD*	*N*1	*N*2	95% lower CI	95% upper CI
Results from Study 1						
Shop signs	24.30	20.60	21	25	15.60	33.00
People	21.60	20.30	23	25	13.10	30.20
Nature	19.50	15.50	22	25	12.90	26.00
Advertisements	17.20	18.00	24	25	9.56	24.80
Other text	5.66	5.87	19	25	3.19	8.14
Traffic	5.37	6.93	19	25	2.44	8.30
Architecture	4.19	5.50	14	25	1.87	6.51
Graphic elements	2.08	3.25	12	25	0.71	3.45
Street signs	0.15	0.42	3	25	0.00	0.32
Result from Study 2						
Shop signs	26.10	9.29	21	21	21.90	30.30
People	22.10	11.50	21	21	16.80	27.30
Other text	12.00	4.86	21	21	9.77	14.20
Traffic	11.20	5.69	20	21	8.57	13.80
Nature	7.39	3.60	21	21	5.76	9.03
Architecture	6.59	6.21	21	21	3.77	9.42
Graphic elements	5.71	3.15	20	21	4.28	7.14
Street Signs	4.11	2.32	20	21	3.05	5.16
Advertisements	3.79	2.49	21	21	2.66	4.93
Animal	1.07	0.89	17	21	0.64	1.50

The Category is sorted by the number of the Mean percentage. *N*_1_ represents the actual number of participants who viewed the given object category. *N*_2_ represents the number of participants who were used to calculate the descriptive statistics. If one participant did not make any fixations on one object category, 0 was included in the calculation of the average percentage and SDs.

#### 2.2.2. Viewing behaviour and aesthetic evaluations toward the target signs

##### 2.2.2.1. Viewing behaviour toward the target signs

Secondly, to test our second research question: ‘How does viewing behaviour correlate with the aesthetic qualities of text?’, we analysed the relationships between the viewing behaviour (i.e., the number of fixations fall into our target signs, viewing time for them) and their aesthetic value, measured in the follow-up test.

**Figure 4:**
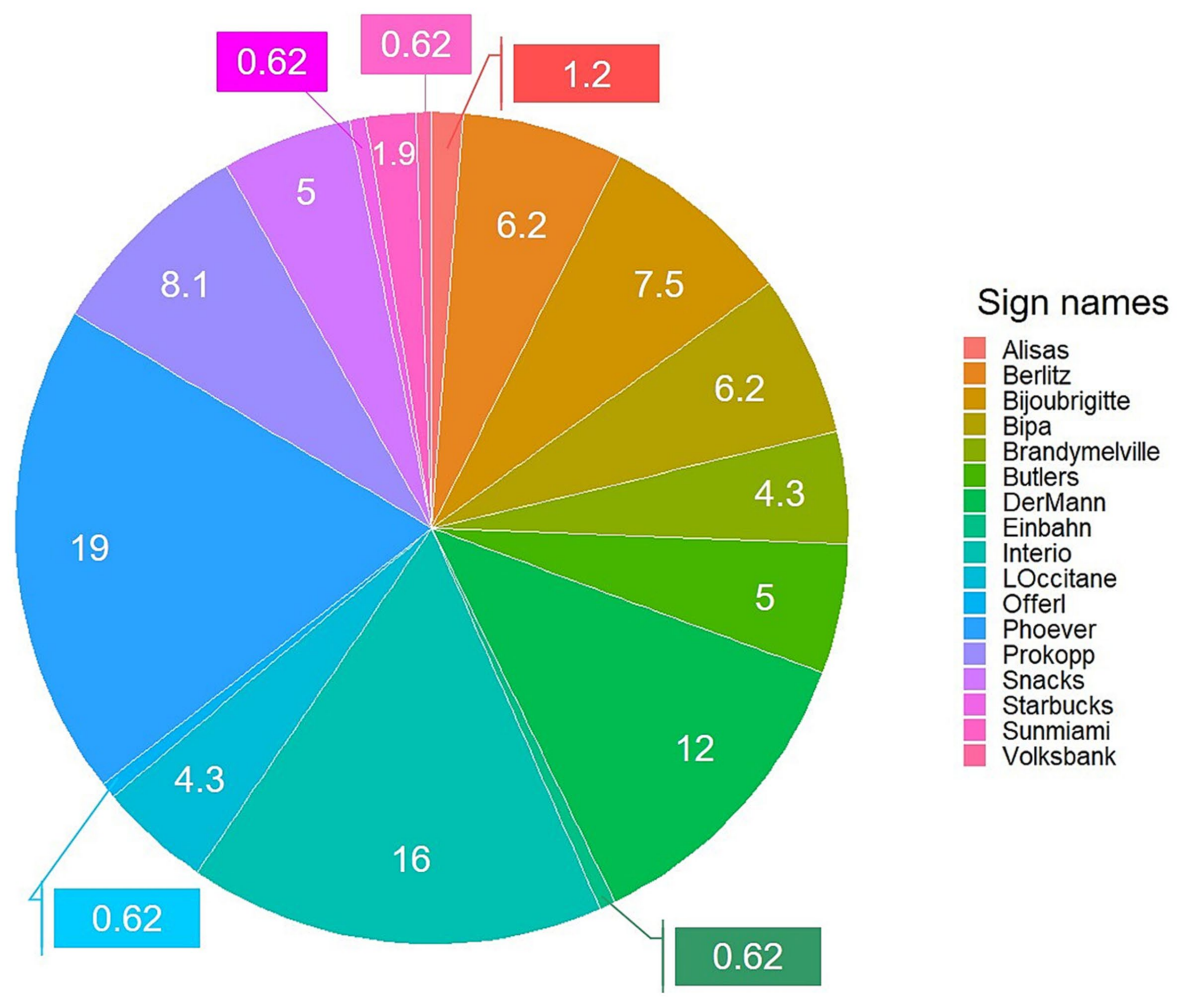
Pie chart using all fixations made by all participants for the 20 target signs from the results of Study 1. The number shown in the chart represents the percentage of fixations that fell into the given sign.

Before assessing the relationship between the viewing behaviour toward target signs and its aesthetic evaluation, we analysed how much attention our 20 target signs actually received in the testing street capture in general, as well as how long each sign was viewed. Overall, 161 fixations fell into the 20 target signs (11.66% out of the total 1,381 fixations), and the average viewing time, measured *via* the frame length, across the 20 target signs was 11.53 (c.a. 380 ms, *SD* = 6.53). The total amount of fixations toward each sign across all participants (%) were calculated (see Figure 4). 47% of the total fixations fell into three signs; *Der Mann* (12%), *Interio* (16%), and *Phoever* (19%), and there were three signs which did not received fixations at all from the participants; *Misttelefon*, *Pizza*, and *To go*.

Further, we computed the average percentage of the fixations as well as viewing time, using individual participants’ data (see Table 3). The average values for percentage of fixations were calculated in the same manner as in the previous section. For the viewing time, first, the average viewing time was computed, using the total duration of viewing times for each sign per participant. For example, when one participant gave a single fixation on one sign, the duration of this fixation was used to calculate the average viewing time across the participants. However, when one participant gave multiple fixations on one sign, the sum of each fixation duration of this participant was used for the calculation. We adapted this method, as our interest was not to assess how long on average the sign was viewed in each fixation. Rather, our main focus was to assess how much one’s attention is held to each sign in total. Note that, as with the computation for the amount of the fixations, when a participant did not give any fixations on one sign, 0 was used for the computation.

**Table 3:** Descriptive statistics for the number of fixations and viewing time per target sign in Study 1.

Sign names	*N*	Percentage of the fixations				Total viewing time (frame length)			
		Mean	SD	Lower CI	Upper CI	Mean	SD	Lower CI	Upper CI
Alisas	2	0.90	3.49	−0.54	2.34	0.84	3.08	−0.43	2.11
Berlitz	8	8.58	21.37	−0.24	17.40	4.64	8.73	1.04	8.24
Bijoubrigitte	4	2.29	5.98	−0.18	4.76	4.56	14.05	−1.24	10.36
Bipa	5	2.44	5.14	0.32	4.56	4.68	12.31	−0.40	9.76
Brandymelville	6	3.36	6.97	0.48	6.24	4.56	13.16	−0.87	9.99
Butlers	7	3.74	7.00	0.84	6.63	3.68	7.53	0.57	6.79
DerMann	12	10.48	15.82	3.95	17.01	8.24	10.96	3.71	12.77
Einbahn	1	0.24	1.18	−0.25	0.72	1.00	5.00	−1.06	3.06
Interio	11	13.91	22.98	4.43	23.40	9.60	16.99	2.59	16.61
LOccitane	5	2.62	5.97	0.16	5.08	3.48	8.06	0.15	6.81
Misttelefon	0	–	–	–	–	–	–	–	–
Offerl	1	0.40	2.00	−0.43	1.23	0.40	2.00	−0.43	1.23
Phoever	14	16.24	19.43	8.22	24.26	14.32	18.49	6.69	21.95
Pizze	0	–	–	–	–	–	–	–	–
Prokopp	9	7.15	11.83	2.26	12.03	5.88	11.07	1.31	10.45
Snacks	5	6.73	21.43	−2.12	15.57	4.92	10.65	0.53	9.31
Starbucks	1	0.24	1.18	−0.25	0.72	0.44	2.20	−0.47	1.35
Sunmiami	2	0.46	1.58	−0.20	1.11	2.36	8.20	−1.02	5.74
Togo	0	–	–	–	–	–	–	–	–
Volksbank	1	0.24	1.18	−0.25	0.72	0.68	3.40	−0.72	2.08

*N* represents the actual number of participants who viewed the given target sign. Lower/Upper CIs represent the lower/upper 95% confidence intervals. For the viewing time, when only one participant gave a single look on the given target sign, NAs were produced for SDs and confidence intervals.

Seeing the actual number of participants who viewed the target sign (see Table 3), no signs were viewed by all of the participants. The most viewed sign on average was *Phoever* (16.24%) by 14 participants, followed by *Interio* (13.91%) and *Der Mann* (10.48%). *Phoever* received the longest viewing time (14.32 frames, c.a. 470 ms), followed by *Interio* (9.60 frames, c.a. 310 ms) and *Der Mann* (8.24 frames, c.a. 270 ms). However, seeing that those signs were viewed by relatively few participants, these results seem to reflect a strong influence of individuals.

##### 2.2.2.2. Aesthetic evaluations X viewing behaviour

**Figure 5:**
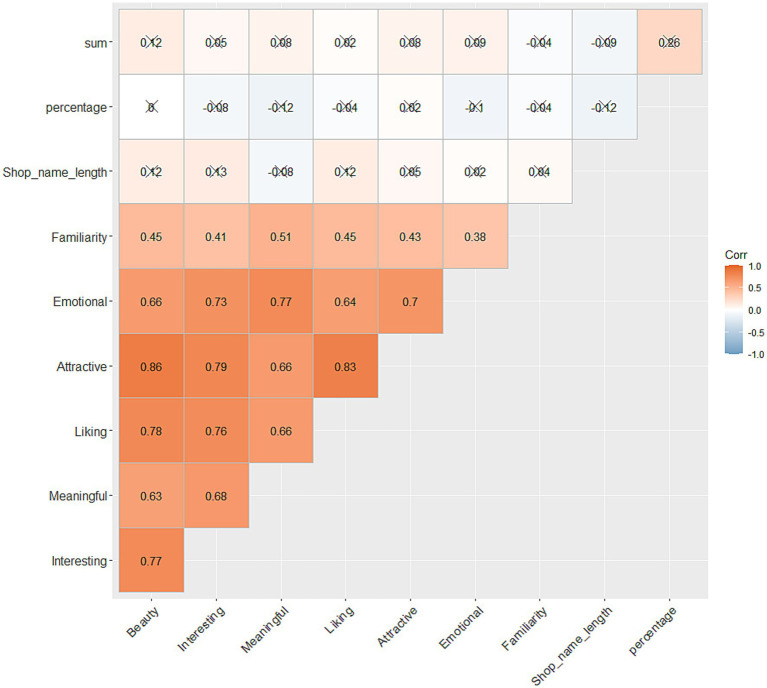
Correlation plot between the divided attention (%), total amount of viewing time, character length and aesthetic ratings toward signs. The numbers in the figures show the actual scores of Kendall’s Tau. *X*s in the figure represent where the results of the correlation tests were not significant.

To assess the relationship between aesthetic ratings for the signs and viewing behaviour, correlation scores were computed between each of the aesthetic ratings for each target sign, sign character length, divided attention toward the target sign in percentage as well the total duration of viewing time, measured *via* frame length. As all rating scores were not normally distributed (as was visually assessed before the analysis), Kendall’s Tau scores were computed. Figure 5 shows the correlation plot for all variables listed above. Note that, for the computation of the correlation scores, the data points from the participants who are missing parts of rating scores, due to recording issues, were not used. For the evaluation of the results from 36 correlation tests, we used adjusted alpha level *p* = 0.0011 (0.05/45). The correlation scores as well as the correlation matrix shown in Figure 5 are made with *cor_pmat*() and *ggcorrplot*() functions in rstatix (Kassambara, 2022) and ggplot2 (Wickham, 2016) packages, respectively.

As can be seen in Figure 5, no systematic relationships were found between viewing behaviour, aesthetic ratings for target signs, and the number of characters in target signs. However, all of the aesthetic ratings toward the signs were positively correlated. This point will be further discussed in the discussion. Hence, our second hypothesis: more aesthetically valued texts also yield longer fixation times, was not supported.

#### 2.2.3. Aesthetic evaluations X memorability (recognition task performance)

The third research question we aimed to address is whether memorability is driven aesthetic evaluations (e.g., do people recognize target signs better if they are beautiful?). The data revealed that, overall, participants correctly responded ‘yes’ and recognized the target signs 48.47% of the time. Participants correctly responded ‘no’ and rejected the distractors 96.52% of the time. Participants’ overall performance was better for detecting the distractor signs than for the target signs, but performance was not greater than chance for detecting the signs from the eye-tracking path. However, it is noted this also does not follow the assumption that all participants included in the analysis viewed the target signs during the eye-tracking walk, since many signs received little or no fixations (see Table 3).

To predict the probability of memory performance as a function of the individual rating scales, we performed a series of multinomial logistic regression models, using each rating scores as independent variables, and four response types in the memory recognition task (i.e., False Alarm, Correct rejection, Hit, Miss) as the dependent variable. Note that, amongst the four categorical responses in the recognition task, False Alarm was set as a baseline for the prediction of odds ratios. The results of all models are shown in Table 4. For building and running multinomial logistic regression models, we used *multinom*() function in nnet package (Venables and Ripley, 2002).

**Table 4:** Multinomial logistic regression models using aesthetic rating scores as predictors for the odds ratio of memory recognition responses.

**Characteristic**	CR			Hit			Miss		
	**OR**	**95% CI**	***p***	**OR**	**95% CI**	***p***	**OR**	**95% CI**	***p***
Beauty	0.95	0.82, 1.12	0.60	1.29	1.09, 1.51	0.0020	0.85	0.73, 1.01	0.06
Interesting	1.04	0.90, 1.21	0.60	1.45	1.24, 1.70	<0.001	0.98	0.84, 1.15	0.80
Meaningful	0.86	0.74, 1.00	0.04	1.52	1.30, 1.76	<0.001	0.94	0.81, 1.09	0.40
Liking	0.96	0.83, 1.12	0.60	1.33	1.14, 1.56	<0.001	0.90	0.77, 1.05	0.20
Attractiveness	0.93	0.81, 1.08	0.40	1.26	1.08, 1.46	0.0030	0.85	0.73, 0.99	0.04
Emotional	0.97	0.83, 1.13	0.70	1.43	1.22, 1.67	<0.001	0.97	0.83, 1.14	0.70
Familiarity	0.64	0.57, 0.72	<0.001	1.52	1.30, 1.76	<0.001	0.68	0.60, 0.77	<0.001

OR, Odds Ratio; CI, Confidence Interval. For the evaluation of the results from seven models, we used adjusted alpha level *p* = 0.007 (0.05/7).

The results indicate that across all ratings, the probability of a hit response increased significantly as the rating scores increased. In other words, when signs were more beautiful, interesting, meaningful, etc., participants answered ‘yes’ to recognising the target stimuli in the memory task. However, except for familiarity ratings, this trend was not found for correct rejection and miss responses. For familiarity, the rating scores influenced all response types. Specifically, when participants were more familiar with the sign, the probability of a hit response increased, and the probability of a correct rejection and miss response significantly decreased. This can be interpreted as a tendency to answer ‘yes’ to the presence of a sign in the testing environment regardless of stimulus type (target/distractor), when the signs are more familiar to the participants.

#### 2.2.4. Viewing behaviour X memorability (recognition task performance)

As viewing behaviour during the walk might also impact on memory performance (e.g., when the participants looked longer at a sign, they might remember this sign better, regardless of its aesthetic quality), we perform a follow-up analysis, predicting the probability of memory performance as a function of total amount of viewing time. Note that, in the present experiment (Study 1), total frame length was used as a proxy of the viewing time as with other analyses. As the viewing time can only be obtained for the target signs, the possible response in the memory test was either Hit or Miss. Since we have binary output, we used logistic regression model, using the frame length as the independent variable, and two response type (Hit, Miss) as the dependent variable. Note that, in the actual code, Hit was coded as 1, and Miss as 0. The results of this regression model suggested that, the actual viewing times toward the signs did not predict the recognition task performance (OR = −0.002, 95%CI [−0.0002, 0.02], *p* = 0.853).

### 2.3. Discussion

The first aim of Study 1 was to address the prevalence of reading experiences within the visual exploration of a street setting. Indeed, it was found that relative to other viewing categories, text and specifically shop signs were looked at the most. However, our results also suggest that, amongst text elements, participants divide their attention differently. Specifically, some categories with texts received far less attention compared to the Shop signs (e.g., 11% for Advertisements and 0.22% for Street signs), suggesting it was not that participants pay attention to texts in a natural environment in general but rather selectively paid more attention to the Shop signs. As aforementioned in the introduction, though paying attention to the traffic situation for better locomotion is one of the major tasks in urban street environments, interestingly, the participants paid little attention to the street signs.

Whilst this is a novel and compelling finding, it appears somewhat plausible that substantial viewing behaviour would be dedicated toward shop signs, nature and people due to the attributes of the street. The testing street, Mariahilferstraße, is a main shopping street in the city of Vienna, and, as such, there is, as one may expect for a street of this kind, a huge density of signs. Consequently, it is not just that signs capture attention, but there is a certain expectation that they ought to, given there are a lot of signs available and the main purpose of the street involves shopping. Thus, further investigation on an alternative urban street, with perhaps a more ‘normal’ balance of signage, would be important to form a solid conclusion of reading prevalence in such settings.

Our second aim was to assess how viewing behaviour correlates with the aesthetic qualities of text. It was hypothesised that the more aesthetically valued texts also yield longer fixation times, however no systematic relationships were found. This somewhat unexpected result may allude to the fact that multiple types of stimulation may draw visual attention and this led to visual engagement. That is, while aesthetically pleasing stimuli (i.e., attractive, beautiful, liked, etc.) can incite engagement, this can also arise through other types of stimulation (e.g., aversion, confusing, threatening, etc.). However, this point should be, in part, further addressed with improved study design. Specifically, as shown in Supplementary Table S4, the aesthetic rating scores for each sign did not scatter, especially toward the higher values; i.e., many participants did not choose the values ‘6’ or ‘7.’ This trend was also visually obvious when the normality was evaluated in the aesthetic rating data before the computation of the correlation scores. In other words, in this study, we lacked signs that the participants evaluated highly across any scales, meaning the relationship between the viewing behaviour and aesthetic ratings was not fully captured. We would like to note that such difficulties in selecting stimuli/manipulating some specific values can happen especially in the field environment, where we cannot fully control the contents of the environment. Such limitations found in field environment should/could be covered by follow-up testing, perhaps in well-controlled laboratory studies. This point will be further addressed in the General Discussion together with the results of Study 2. It should be noted, though there was no systematic relationship between the viewing behaviour and aesthetic ratings, the aesthetic ratings themselves measured in the follow-up session were positively correlated. This can be interpreted that many scales included in the present study acted quite similarly. Based on this finding, for Study 2, we tried to reduce the number of scales. To this aim, we performed PCA (principle component analysis) for the purpose of reduction of dimensions. The results showed that three principle components explained around the 90% of the proportions of variance in the aesthetic rating data. Those three components could be divided into an appraisal dimension (e.g., beauty, liking, attractiveness), a meaningfulness dimension (i.e., meaningfulness), and a familiarity dimension (i.e., familiarity). The detailed results as well as the code for this part of result can be found in Supplementary material (OSF). Based on this result, we decided to use only three scales in the follow-up session for the aesthetic evaluation, namely, beauty, meaningfulness, and familiarity. Beauty was explicitly chosen amongst the other scales, as it tends to relate highly to other hedonic assessments such as liking or interest, serving as a general measure of aesthetic appreciation especially amongst lay people (Jacobsen et al., 2004).

Lastly, we investigated whether aesthetic texts are more memorable. Looking at the recognition data alone, we found participants’ overall memory performance was not better than chance. Nevertheless, when compared with aesthetic evaluations of the texts, participants’ memory for target signs increased as ratings increased. Though, this may be due to a response bias, saying yes in the recognition task, irrespective of the text sign’s actual presence. It is, however noted that for the familiarity ratings, memory performance improved significantly across all possible responses to the task, so that participants were not only better at correctly identifying the target signs that were more familiar, but also better at correctly rejecting the distractor signs too. These results suggest the aesthetic quality of texts does indeed drive memory in some way, with familiarity having the strongest influence on memorability.

Overall, especially our first and third hypotheses seem to be supported from the results of Study 1. However, as discussed, such results can stem from specific characteristics of the street environment or expectations from participants. To test the generalizability of our findings further, we conducted another field study in a quite different street environment in Study 2.

## 3. Study 2

### 3.1. Materials and methods

#### 3.1.1. Participants

A sample of 26 participants (19 female, 6 male, 1 other; *M*_age_ = 25.96, *SD*_age_ = 7.72, range: 18–46 years) were recruited with a circular email sent out University of Vienna. All participants included in the analysis had normal vision and spoke German. The average height of the participants was 169.92 cm (*SD* = 10.79, range: 150–192 cm). Anonymity was guaranteed with the use of personal identifiers and participants received monetary compensation (10 €). The study corresponds with the ethical standards of the Declaration of Helsinki and the ethical regulations at University of Vienna.

#### 3.1.2. Apparatus

The Tobii Pro Glasses 3 wearable eye-tracking headset was utilised for the purpose of the study. We note there was a change in eye-tracking glasses from Study 1 due to equipment availability, and also given that this device and compatible software would facilitate the speed of the time-consuming manual coding process. The Tobii portable glasses (76.5 grams) are embedded with eight infrared illuminators and two eye cameras per eye, as well as a scene camera to recorded the outer environment with 106° view in 16:9 format (H: 95°, V: 63°; 1920 × 1,080 at 25 fps, 50 Hz). The eye-tracking data was collected on a connected recording unit (312 grams) fitted with an SD card, but controlled on an Android smartphone, running the Glasses 3 Controller app.

Calibration was administered with a target marker on a calibration card. This requires participants to focus on the centre of the calibration card, with the card placed approximately 1 m distance away at eye-level (area where we require most accuracy).

#### 3.1.3. Setting

**Figure 6:**
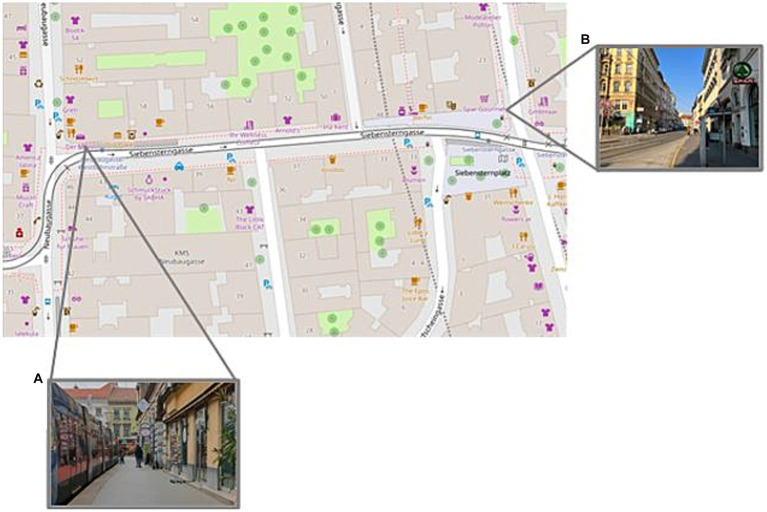
OpenStreetMap image, depicting the eye-tracking route along Siebensterngasse 42 to 60 in Vienna’s seventh district. The four images denote the start **(A)** and finish **(B)** positions along the eye-tracking path.

A 180 m section of Siebensterngasse in the 7th district of Vienna was chosen for the eye-tracking path. The start point was set at Siebensterngasse 42 (48.20255, 16.35150; 6,922 + WJ Vienna) and the end point was set at Siebensterngasse 60 (48.20239; 16.34927; 682X + VP Vienna) (Figure 6). This street sits behind the back of the MuseumQuartier and this particular section joins from the popular shopping street Neubaugasse. There are a range of retail stores, restaurants and other services along this street. Whilst this is not a pedestrianised street, with a road and 49 tram line in use, only one small side street (Stuckgasse) with little vehicle use fell within this path. This meant there would be minimal traffic interfering with the eye-tracking path. Participants walked west to east and back again. All recordings took place between 7:30 am – 11:30 am when the street was less busy and the majority of stores were still closed or less frequented.

#### 3.1.4. Eye-tracking procedure

The eye-tracking testing procedure was the same as Study 1. To keep the conditions across participants as similar as possible and avoid too many changes to the street, data was collected in a short time span of 1 week. Testing occurred across all days within this time (Monday to Sunday) to cover slight differences in the environment across the span of a week. For the most part, the general street setting was the same, with minor changes to traffic (i.e., number of trams, passers-by, waste collection) and one occasion where maintenance work was carried out to a drain at the starting point of eye-tracking path (see Supplementary Table S1).

#### 3.1.5. Follow-up session procedure

The follow-up session procedure was the same as Study 1. Again, 20 photographs were taken of text elements from the eye-tracking path and 20 distractor photographs containing text from other areas within the city streets (see footnote 4). All photos were taken on a mobile phone camera (Huawei Mate40 Pro) and cropped to a 16:9 aspect ratio with landscape orientation. Photos portrayed text from shop fasciae/signs and were selected at random from a larger corpus of images photographed from both directions and both sides of the street. Though restricted to the text elements present within the testing environment, we chose to focus on text signs due to the high prevalence and variation of shop signage within the street environment. We also reported such variations in terms of physical features (e.g., size, height, position, number of characters) (see Supplementary Table S2).

Participants completed a further questionnaire 1 week after the initial in-person eye-tracking session. Firstly, participants performed an open recall task, followed by a recognition and rating task for the 40 photographs provided (20 from the eye-pathing path, 20 distractors). For the recognition task, participants indicated whether the photographs were of text signs that were present on the eye-tracking path (yes/no dichotomous response). Participants also rated the 40 photographs for subjective aesthetic judgement on 7-point scales (beauty, meaningfulness, familiarity). Finally, participants completed the LHQ v3.0 (Li et al., 2020), in which they reported demographics and subjective ratings about their language background. Again, the open responses to the memory recall as well as the measurements of LHQ v3.0 questionnaire were performed for exploratory purpose for planning our future studies, and they are beyond the scope of the present study. Hence, analyses for those measures will not be presented in the present study.

#### 3.1.6. Data preparation

First, we checked the general quality of the eye-tracking data obtained by the 26 participants. For the eye movement data, five participants were excluded due to insufficient data quality, as calculated by a marked reduction in the percentage of fixation data collected compared to the amount of expected data. We note a possible explanation for this reduction in data quality could partly be due to the lighting conditions in the field environment, where, during the mornings, there was sometimes strong sunlight (see Holmqvist et al., 2023). The data for the remaining participants with complete eye-tracking video material was included for this analysis; *N* = 21 (13 female).

Overall, 14,361 fixations were made in entire recording. The number of fixations made by individual participants ranged from 87 to 952. The total time of the annotated videos was 1 h 57 min 20 s, Regarding the number of the fixations longer than 200 ms, overall, there were 6,621 fixations. We again note only the fixations longer than 200 ms were included in the main analysis below. The annotations (see Table 1) and data extraction were performed with iMotions software (version 9.3), and the eye-tracking data was then analysed with the same R environment as in Study 1.

### 3.2. Results

We again calculated the average scores for information regarding the eye-tracking street location for the 26 participants prior to the main part of the analysis, including participants’ liking (*M* = 4.46, *SD* = 0.65), familiarity (*M* = 3.00, *SD* = 1.65) and frequency (*M* = 2.23, *SD* = 1.18) of visiting this section of the street. Further, we asked if participants would visit this part of the street again (No = 11, Yes = 15). The scores for comfort of the glasses were also assessed on a 5-point scale (1 = not at all, 5 = very comfortable). The average score for this question was 4.15 (*SD* = 0.88). Thus, it was again assumed that the participants’ viewing behaviour was not constrained by wearing eye-tracking glasses and could be included in the analysis.

#### 3.2.1. The number of fixations per object category

Firstly, to see which objects captured attention overall, again, the total amount of fixations toward each category across all participants (%) were calculated (see Figure 2B). 59% of the total fixations fell into Shop Signs (26%), People (21%), and Other Text (12%). The remaining 30% of total fixations fell toward Other Text (12%), Traffic (11%) Nature (7%), Architecture (6.5%), Graphic Elements (6.2%), Hence, this result shows, even with less amount of shops, Shop signs are by far a dominant component that people pay attention to amongst other (text) categories while walking regardless of the testing environments. Though Nature was one of the dominant categories to be viewed in Study 1, only 7% of total fixations fell into this category in Study 2. Such difference seems to reflect the different characteristics of the two streets, as in Siebensterngasse, there are fewer green components compared to the Mariahilferstraße.

Further, the percentage of the fixations on each category per participant is shown in Figure 3B, and the descriptive statistics for this measure is presented in the bottom part of Table 2, showing the variance in participants’ gaze distribution. Descriptive statistics were calculated in the same manner as in Study 1. As with the results from Study 1, People and Shop signs were the object categories to which the participants made the most fixations. Hence, our second study, in a quite different street environment, showed similar results as Study 1.

#### 3.2.2. Viewing behaviour and aesthetic evaluations toward the target signs

##### 3.2.2.1. Viewing behaviour toward the target signs

Next, the relationships between the viewing behaviour (i.e., the number of fixations that fall into our target signs, viewing time for them) and their aesthetic value, measured in the follow-up test, were analysed.

**Figure 7:**
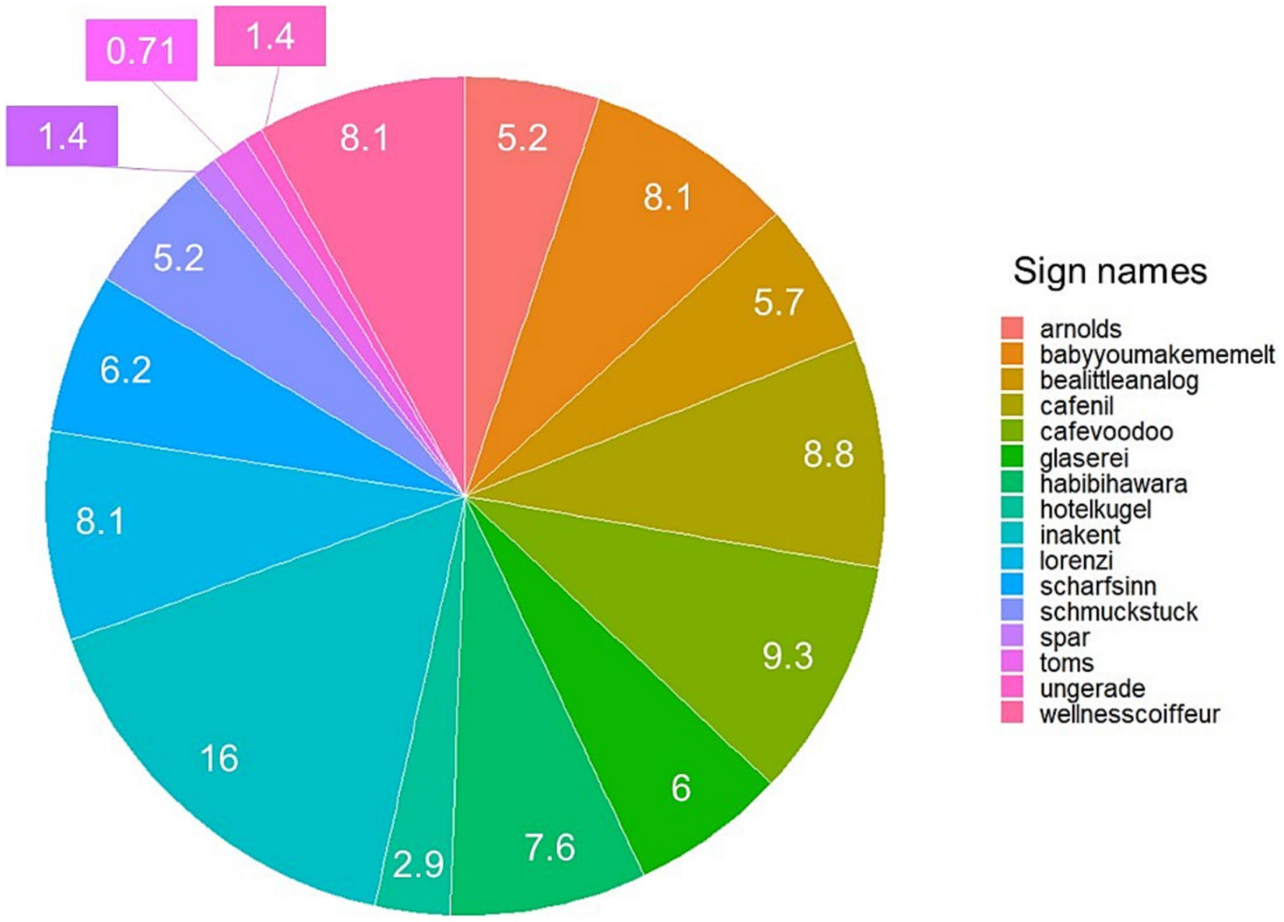
Pie chart using all fixations made by all participants for the 20 target signs from the results of Study 1. The number shown in the chart represents the percentage of fixations that fell into the given sign.

Overall, 420 fixations fell into the 20 target signs (6.34% out of the total 6,621 fixations), and the average viewing time across all 20 target signs was 240.77 ms (*SD* = 311.10). To assess which signs were viewed the most and least, the total amount of fixations toward each shop sign (%) were calculated (see Figure 7). The most viewed sign was *Ina Kent* (16%), followed by *Café Voodoo* (9.3%) and *Café Nil* (8.8%). There were four signs (*adlerhof*, *chicohangematten, r_s, tabaktrafik*) that received no fixations.

We also computed the average percentage of the fixations as well as viewing time, using individual participants’ data (see Table 5). The average values for percentage of fixations as well as the average viewing time were computed as in the same manner in Study 1.

**Table 5:** Descriptive statistics for the number of fixations and viewing time per target sign in Study 2.

Sign names	*N*	Percentage of the fixations				Viewing time (frame length)			
		Mean	SD	Lower CI	Upper CI	Mean	SD	Lower CI	Upper CI
adlerhof	0	–	–	–	–	–	–	–	–
arnolds	17	5.32	5.18	3.18	7.46	615.92	374.96	377.68	854.15
babyyoumakememelt	11	3.89	5.95	1.44	6.35	1580.90	821.06	993.55	2168.25
bealittleanalog	17	3.55	3.75	2.00	5.10	1477.67	1400.42	587.89	2367.45
cafenil	17	11.83	11.17	7.22	16.44	944.85	683.79	531.64	1358.05
cafevoodoo	16	5.41	5.70	3.06	7.77	1389.29	850.13	898.43	1880.14
chicohangematten	0	–	–	–	–	–	–	–	–
glaserei	14	3.42	3.64	1.91	4.92	651.85	370.86	427.74	875.95
habibihawara	16	4.43	4.56	2.55	6.31	1571.17	1518.45	606.39	2535.94
hotelkugel	12	1.60	2.24	0.67	2.52	619.50	353.69	323.81	915.19
inakent	19	16.49	12.81	11.20	21.78	1590.83	1265.07	961.73	2219.94
lorenzi	16	4.58	5.36	2.37	6.79	1157.20	1168.96	509.85	1804.55
r_s	0	–	–	–	–	–	–	–	–
scharfsinn	16	3.58	3.80	2.01	5.15	1308.86	836.60	825.82	1791.89
schmuckstuck	13	3.10	3.44	1.68	4.52	989.82	1209.42	177.32	1802.32
spar	8	0.86	1.54	0.23	1.50	371.67	181.88	−80.16	823.49
tabaktrafik	0	–	–	–	–	–	–	–	–
toms	10	1.49	2.67	0.39	2.60	330.00	60.00	255.49	404.51
ungerade	8	1.13	2.05	0.28	1.97	468.33	140.43	119.49	817.18
wellnesscoiffeur	18	5.32	4.86	3.31	7.33	948.00	681.15	536.39	1359.61

*N* represents the actual number of participants who viewed the given target sign. Lower/Upper CIs represent the lower/upper 95% confidence intervals.

Compared to Study 1, more participants gave fixations to the target signs in Study 2 (see Table 2). This difference might be caused by the number of available shop signs in the testing environment; whilst there are numerous shop signs available in the Mariahilferstraße, there are less shops in the Siebensterngasse. Hence, when the participants viewed any signs, the chance that our target sign received looks should be generally higher. However, again, no signs were viewed from all of the participants. The most viewed sign on average was *Inakent* (16.49%) by 19 participants, followed by *Café nil* (11.83%) and *Arnolds* and *Wellness coiffeur* (both 5.32%). *Inakent received* the longest average total viewing time (1590.83 ms), followed by *Baby youmakememelt* (1580.90 ms) and *Habibihawara* (1571.17 ms).

##### 3.2.2.2. Aesthetic evaluations X viewing behaviour

**Figure 8:**
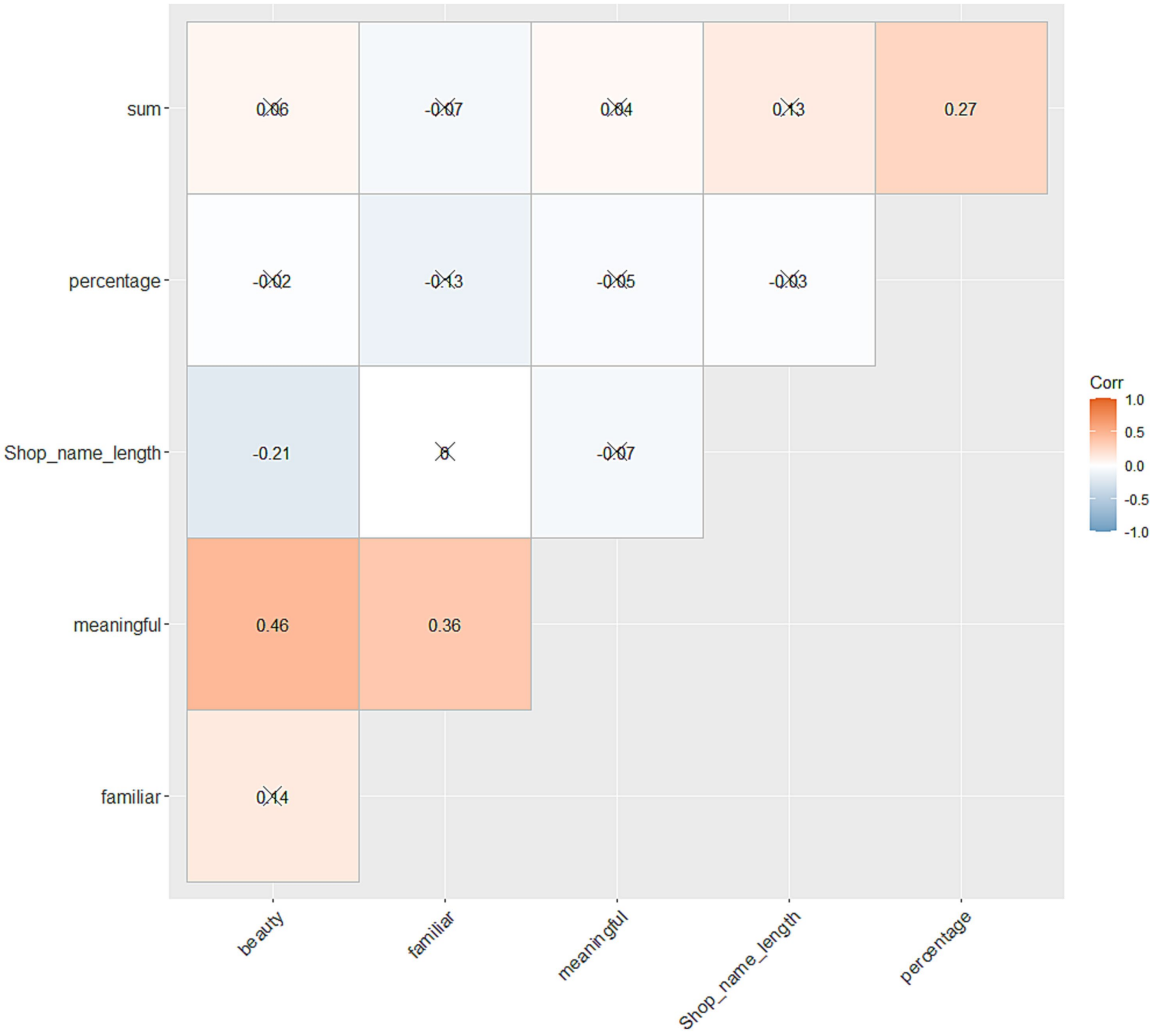
Correlation plot between the divided attention (%), total amount of viewing time, character length and aesthetic ratings toward signs. The numbers in the figures show the actual scores of Kendall’s Tau. *X*s in the figure represent where the results of the correlation tests were not significant.

To assess whether aesthetic value modulates viewing behaviour, a series of correlations were performed between viewing behaviour [i.e., divided attention toward target sign in percentage as well as total viewing time (ms) for each sign per participant], each of the aesthetic ratings and sign character length. Again, as all rating scores were not normally distributed (as was visually assessed before the analysis), Kendall’s Tau was computed. Figure 8 shows correlation plot for all variables listed above. Note that, for the evaluation of the results from 10 correlation tests, we used adjusted alpha level *p* = 0.005 (0.05/15). As in Study 1, no systematic relationships were found between viewing behaviour and aesthetic ratings for target signs. Again, all of the aesthetic ratings toward the signs were positively correlated. One result which is different from Study 1 was the correlation between the divided attention toward the signs and total viewing time toward them. In Study 2, there is a positive correlation between the two variables, meaning when the participants took more looks at the sign, the total viewing time toward the sign increased. This result is quite intuitive, and raises a question why we did not find such relationship in Study 1. Further, Study 2 did find a negative correlation between beauty and sign character length, meaning when texts included more characters, they were evaluated as more beautiful. These points will be further discussed in the General Discussion.

#### 3.2.3. Aesthetic evaluations X memorability (recognition task performance)

The data revealed that, overall, participants correctly responded ‘yes’ and recognized the target signs 18.46% of the time. Participants correctly responded ‘no’ and rejected the distractors 80.96% of the time. Participants’ overall performance was not greater than the chance for detecting the signs from the eye-tracking path. Further, it is noted that there appears to be a general tendency to indicate a ‘no’ response across signs, resulting in a high proportion of correct rejections of distractors and misses of target signs.

Again, to predict the probability of memory performance as a function of the individual rating scales, a series of multinomial logistic regression models were performed. The model structures were the same as in Study 1. The results of all models are shown in Table 6. The results indicate that across all ratings, the probability of a correct rejection and miss decreased significantly as rating scores increased. In other words, when signs were more beautiful, meaningful or familiar, participants tended to answer ‘yes’ for distractor stimuli in the memory task.

**Table 6:** Multinomial logistic regression models for aesthetic rating scores (characteristic) and memory recognition responses.

**Characteristic**	CR				Hit				Miss		
	**OR**_**1**_	**95% CI**_1_	***p***		**OR**	**95% CI**	***p***		**OR**	**95% CI**	***p***
Beauty	0.75	0.66, 0.86	<0.001		0.87	0.73, 1.03	0.11		0.69	0.60, 0.79	< 0.001
Meaningful	0.60	0.52, 0.68	<0.001		1.01	0.87, 1.18	0.90		0.58	0.67	< 0.001
Familiarity	0.48	0.43, 0.55	<0.001		0.96	0.84, 1.09	0.50		0.48	0.42, 0.54	<0.001

1 OR, odds ratio; CI, confidence interval. For the evaluation of the results from three models, we used adjusted alpha level *p* = 0.016 (0.05/3).

#### 3.2.4. Viewing behaviour X memorability (recognition task performance)

As with Study 1, we assessed the impact of viewing times toward the signs on recognition task performance. To this aim, we performed the same structured regression model, using the viewing time (ms) as the independent variable, and two response types (Hit, Miss) as the dependent variable. We note that, while we used the total frame lengths as the proxy of the viewing time, in Study 2, we directly used the viewing time (ms) as the independent variable. Again, the results of this regression model suggested that the actual viewing times toward the signs did not predict the recognition task performance (OR = −0.00, 95%CI [−0.02, 0.0004], *p* = 0.499).

### 3.3. Discussion

The aim of Study 2 was to further investigate attention toward signs in a second location, to see whether the suggested prevalence of text viewing withstands. It was indeed found that even on a relatively ‘normal’ inner-city street, not as inundated with large shop signs, text elements, and specifically shop signs, attracted attention more than other object categories. Our results also suggest in this street environment participants again divide their attention differently to text elements. Some categories with text received far less attention compared to Shop signs (e.g., 12% for Other Text and 4.2% for Street signs), which again suggests it was not that participants pay attention to texts in a natural environment in general, but rather selectively paid more attention to the Shop signs. Thus, with text signs capturing attention on this alternative urban street, this alludes to a high reading prevalence in such settings overall.

Our second aim was to assess how viewing behaviour correlates with the aesthetic qualities of text. It was hypothesised that the more aesthetically valued texts also yield longer fixation times; however, again, no systematic relationships were found. Yet again, this point should be further addressed with improved study design. Specifically, as shown in Supplementary Table S5, the aesthetic rating scores did not scatter largely, especially toward the high values, i.e., many participants did not choose values ‘6’ or ‘7.’ This trend was also visually obvious, when we evaluated the normality in the aesthetic rating data before the computation of the correlation scores. In other words, in this study, we lacked the signs that participants evaluated highly across any scales, meaning the relationship between the viewing behaviour and aesthetic ratings were not fully captured. Moreover, this again could reflect in that types of stimulation other than aesthetic appeal, such as aversion, can incite engagement.

Lastly, we investigated whether aesthetic texts are more memorable. Looking at recognition data alone, we found participants’ overall memory performance was not greater than chance. However, this may be due to a response bias, with a high propensity to answer ‘no’ in the recognition task, therefore, we can question how well this indicates their memory for the signs. Nevertheless, when compared with aesthetic evaluations of the texts, participants’ memory for target signs increased as ratings increased. These results suggest the aesthetic quality of texts does indeed drive memory in some way, with familiarity having the most influence on memorability.

Overall, our first and third hypotheses appear to be supported by the results of Study 2. This suggests our findings stem from different characteristics of the street environment or expectations from participants. In this light, the findings are further discussed in the General Discussion.

## 4. General discussion

The aim of this research was to investigate the way in which people engage with and explore text in an urban setting. In an everyday street context, there is an abundance of text that could capture attention amongst a broader selection of objects present in such a busy, outdoor environment. Thus, an exploratory approach was taken to examine/provide insight into interactions with text in public spaces. Particularly, focus was placed upon which objects are looked at often, what text captures most attention and how viewing behaviour then relates to aesthetic evaluations, and also to memorability.

### 4.1. Viewing behaviour: which objects are looked at most?

The initial query this paper addresses is which objects capture attention. It was hypothesised that signs and texts would receive more attention than other visual objects within the testing street. The fixations recorded from eye-tracking data were assigned to object categories to reveal how much something was looked at. By comparing the distribution of fixations across these categories, both Studies 1 and 2 found shop signs are the components in urban streets that capture most attention across people. This corroborates with previous findings based in laboratory settings that there is an inclination to look at text in real world scenes (Cerf et al., 2009; Wang and Pomplun, 2012). We see this finding despite the noticeable differences between the two study locations here. For example, the greater number of fixations toward traffic and street signs for Study 2, and to nature and advertisements in Study 1, greatly reflects on the street composition. Therefore, this outcome is particularly compelling given that this insinuates similarity in how people divide their attention and read text during such walks, even with such complex settings and despite individual differences.

Further, aside from shop signs, both studies found a high proportion of viewing behaviour toward people, and in Study 1 also to nature. These findings appear plausible given that past research demonstrated a preference (to attend) toward people/faces (e.g., Rousselet et al., 2003; Bindemann et al., 2005; Fei-Fei et al., 2007; Ro et al., 2007; Fletcher-Watson et al., 2008; Langton et al., 2008; Leder et al., 2010) and to nature (e.g., Biederman and Vessel, 2006; Fei-Fei et al., 2007; Tinio and Leder, 2009). In Study 2, we further separated the eye-tracking data for people, finding that whilst participants were inclined to look closely at faces, a large proportion of gaze was directed toward other parts of the body (i.e., legs, feet) (see Supplementary Table S6). This reflects on previous research more specifically on eye guidance and locomotion in natural scenes, in terms of spatial location and saliency with regard to walking safely. This may be due in part to the social ‘costs’ associated with direct eye contact (Goffman, 2008; Cristino and Baddeley, 2009), but also to avoiding colliding into other people (Jovancevic et al., 2006; Foulsham et al., 2011). Cristino and Baddeley (2009) observed greater fixations to the kerb, a low contrast feature, whilst none toward a flock of birds, a highly salient image feature. This suggests attentional guidance toward the task at hand, since the kerb indicates the separation of the road and pavement, which is imperative for a pedestrian to detect when walking safely along the street (see also Patla and Vickers, 2003; Patla, 2004; Marigold and Patla, 2008). This could possibly also explain the inclination to look at the path and to tree trunks/lampposts as potential landmarks (see Foulsham et al., 2011). Thus, it appears the tendency to look toward people and nature within an urban setting could be a result from preference/social cues or obstacles/hazards that occur while walking along a street. This separation was not initially included in our object category list, which followed Mitschke et al. (2017), but we noticed this tendency during the annotation process. As such, any future studies in this line of research might also be interested in this regard.

### 4.2. Viewing behaviour: inter-individual difference in viewing behaviour in target shop signs

Seeing the viewing time for each target shop sign, there was considerable variance in the average viewing times for the individual shop signs of interest. Additionally, it was apparent that some of these signs were looked at by all participants, while others were seldom looked at or not at all. Such a trend might be challenging when it comes to analysing the results. For example, when we want to investigate the relationship between the viewing behaviour and aesthetic quality in the shop signs, it would be impossible to assess such relationship, if the participants do not take a look at the target signs at all in the testing environment.

One can make possible inferences as to why this is the case, and how this could possibly reflect more on the specifics of the particular streets in question. It is established that the position of the text is imperative for it to capture attention (Wang and Pomplun, 2012). Many of the signs used in the follow up depicted the text on a fascia, however it is not known whether text printed on the fasciae were looked at much less than the projecting/hanging signs protruding from the shop fronts. Furthermore, Kim and Park (2021) found signs placed in a higher position elicited more and longer fixations than signs in a lower position. As such, perhaps knowledge of these streets alludes to the fact that Mariahilferstraße has a larger abundance of retail stores, consequently in this location there is a greater disposition to look at the protruding signs. Siebensterngasse, on the other hand, is a much quieter street with less of an incline, hence gaze is directed more at eye level, as opposed to upward, as was often the case with Mariahilferstraße. The nature of the current studies meant there was not a balanced number of signs differing in terms of fascia/protruding (or even high vs. low), to assess whether there was a significant difference between sign position and viewing times.

For these reasons, a suggestion for future studies is to consider the positions of the target signs, and perhaps also the incorporation of images of the protruding signs, which would have been seen at a similar angle during the eye-tracking walk, instead of images taken of the shop front, which would not have been seen face on (as in our follow-up). Such selection criteria would provide better quality of results and analysis in the field environment.

### 4.3. Aesthetic quality of text signs

Given that aesthetic quality has been associated with longer looks, the current studies explored whether the viewing behaviour toward signs was correlated with participants’ evaluations of them. Specifically, we hypothesised that the more aesthetically valued text signs would yield longer viewing times. However, no systematic relationship was found between the ratings and eye movement patterns in both studies. The lack of association may have arisen because the signs that were present were generally rated quite low (see Supplementary Tables S4, S5), as such it is possible they were not high enough in aesthetic value to elicit longer looks. We note this is a major difficulty in the field study design, since there is a certain lack of control in the selected variables, and therefore stresses the importance of follow-up laboratory studies where signs with more variation (i.e., covering the entire scale) in aesthetic value can be implemented.

Alternatively, it may be the case that something other than the aesthetic qualities measured in these studies drives viewing behaviour in this instance (e.g., saliency, aversion, confusion, threat, etc.). For instance, Bojko (2013) notes that elements in the environment may capture attention due to the “visual prominence of the area,” contrasting to the rest of the environment, perhaps in regard to the colour, shape, size, or other physical characteristics (see also Chatterjee, 2003; Estrada-Gonzalez et al., 2020). Further, more specific linguistic characteristics related to text (e.g., word length, word frequency) could influence viewing time, given that longer, infrequent words may require more cognitive processing (see Rayner et al., 2011). It is also plausible that the location of the stimulus in relation to the wider environment also plays a role in attention to text. It is perhaps not even that these areas stand out *per se*, but that prior knowledge leads to expectations that such areas will contain information of importance (Wang and Pomplun, 2012). The extent to which the visual features or word content influence participants ratings is not clear. For this reason, this study cannot ascertain whether this attention to text is driven more by top-down or bottom-up factors (see also Wu et al., 2014).

In addition, the use of a free-viewing paradigm leaves the question as to what determines viewing behaviour rather open, given that there was no specific purpose/task aside from locomotion to drive attention in a particular direction. To address this, a retrospective thinking out loud task could be administered to gain further insight into participants’ thoughts and therefore possibly why longer fixations were to made toward certain signs (see Mitschke et al., 2017). Moreover, a more specific instruction could be implemented during the eye-tracking procedure. Previous research suggests this would mean a change from explorative to active looking (Janiszewski, 1998). In this light, viewing behaviour could be linked more specifically to visual search, and therefore scanning/reading signs for relevancy/meaning, or even implementing other behavioural metrics – if an individual is more inclined to visit a store when attracted to the signs, and therefore if signs are correlated to consumer behaviour.

### 4.4. Memorability of text signs

The current study also provides insights into how the environment is perceived, by considering the memorability of text that features within this space. Text is an element featured in real world scene studies but in the broader context of the natural environment, where it is present amongst so many other ‘distractions’, it still attracts our attention. Therefore, we can question whether such text signs are memorable, and what may drive this memorability. We hypothesised that aesthetic quality of text would be a contending factor, and found that indeed text signs rated higher were better recognised.

Whilst this finding indicates aesthetically values texts have an effect on memorability, this only partially supports the notion that high aesthetic value leads to longer looks and better memory, given that neither of the two studies found a relationship between viewing behaviour and aesthetic quality. Specifically, whilst we see improved memorability (through recognition performance) of signs with higher aesthetic value, we do not find that these aesthetically valued signs are looked at longer. In this light, whilst in previous studies the aesthetic appeal draws visual attention, perhaps confusing or even unappealing elements are equally salient to the visual system (see Wu et al., 2014). Thus, signs eliciting aversion could also be retained in memory, given that arousal -irrespective of valence- could predict memorability. Further, we also find that not all participants looked at all of the target signs in the testing streets, therefore one can question how much the memory performance for signs reflects on participants’ actual engagements with text during the eye-tracking walk. It is possible that we highlight upon a tendency to report recognising a sign when in fact the participant did not look at it. We can infer that this is due to prior knowledge of the street and familiarity with the text signs that are present. Brockmole (2008) notes the memory advantage for objects which are linked to the setting in which they were shown. This insinuates that there is a stronger memory trace for aesthetically valued signs, but this memory trace may be built over time and not from a single walk of around 5 min where it was possible to read such signs. To see if this is the case, and that attending to aesthetically valued signs during a natural walk lead to better memory, this would require selecting participants who are completely new to the surroundings and with no prior knowledge about the street in question.

Further, from these studies it is not clear the extent to which visual features of text drive memory, and it is possible something else might be driving both the aesthetic value ratings and the memorability ratings. As aforementioned, image saliency models for scene viewing suggest there are both image-driven and informational-driven features. In this light, both need to be addressed to assess the interaction between the two. Perhaps the sign that is more memorable is so because of a participants’ likelihood to visit the shop, or the purpose of their walks (to shop, to find food, to commute), and these in turn also lead them to regard such signs with high aesthetic value. Addressing this would require the isolation of such features, also since it is not clear what specifically about the signs leads to this aesthetic/memory relation (e.g., fonts, colors, size, word frequency, word length, etc.).

### 4.5. Limitations

A smaller sample size allowed us to explore viewing behaviour more meticulously across the span of each individuals’ walk, however is a limitation of the current studies, especially since we are dealing with behaviour in such a complex, multifaceted environment with supposedly large individual differences. Despite this, we still see a high proportion of attention toward shop signs/text. Moreover, whilst carrying out the same walk (A to B, B to A) allowed the midpoint environment during the calibration check to remain the same across participants, we also reflect that counterbalancing the start direction of the walk would be prudent to establish a contrasting priming effect. Therefore, it would be interesting to see how similar viewing behaviour is under more controlled settings, such as within a laboratory context or using virtual reality simulations, as this would mean all participants experience the same street conditions and subsequently have the same likelihood of viewing text elements. Further, this would combat another limitation of this study in that, given the laboriousness of manually annotating mobile eye-tracking data, it was not feasible to quantify the number of potential opportunities for the individual participants to have seen the signs and proportion this to the actual incidents where they looked at the signs.

Whilst both Study 1 and 2 uncover compelling findings regarding text perception and the prevalence to look at signs, a direct comparison cannot be made for the viewing behaviour patterns across these two locations. The different hardware and software used in both studies, whilst allowed for more robust measure of eye-tracking performance for the latter study, means that it is not possible to determine if differences in results arise due to the methods of data collection (e.g., differences in viewing behaviour and fixation durations across both studies due to the devices’ sampling rates, temporal resolution, spatial accuracy, etc.) or if this reflects the nature of viewing behaviour in the two testing environments. For example, the attention of participants in the first study was short and scattered, in contrast to the second street where there were generally longer fixations. This may be due to the first street having much more signage and other distractors competing for attention, as compared to the second with less available stimuli and a quieter atmosphere. Yet it is also possible that this reflects not on the viewing behaviour of the testing environment, but that performance of fixation detection differed between the two eye-tracking devices. However, this remains speculative and cannot be confirmed with direct statistical comparisons given that the possible differences in data quality cannot be excluded. We would suggest that future studies comparing locations use the same devices and software so that differences in fixations can be further elucidated.

To sum, these studies reveal how attention is divided during a natural walk, finding that indeed shop signs and text elements do attract people’s attention in an urban street environment. We also address caveats that arose and propose improvements to consider in future studies, with the hope that this aids future research on natural reading behaviours and aesthetic viewing in urban environments.

## Data availability statement

The datasets presented in this study can be found in online repositories. The names of the repository/repositories and accession number(s) can be found at: https://osf.io/ajr48/.

## Ethics statement

Ethical approval was not required for the studies involving humans because ethical review and approval was not required for the study on human participants in accordance with the local legislation and institutional requirements. The studies were conducted in accordance with the local legislation and institutional requirements. The participants provided their written informed consent to participate in this study.

## Author contributions

KC and HL contributed to the design of the study. KC and AS were responsible for data collection. KC and JM analysed and interpreted the data. KC, JM, and HL drafted the manuscript. All authors contributed to the article and approved the submitted version.

## Funding

This project has received funding from the European Union’s Horizon 2020 research and innovation programme under the Marie Skłodowska-Curie grant agreement no. 860516. JM was supported by the Vienna Science and Technology Fund (WWTF) [10.47379/ESR20034].

## Conflict of interest

The authors declare that the research was conducted in the absence of any commercial or financial relationships that could be construed as a potential conflict of interest.

## Publisher’s note

All claims expressed in this article are solely those of the authors and do not necessarily represent those of their affiliated organizations, or those of the publisher, the editors and the reviewers. Any product that may be evaluated in this article, or claim that may be made by its manufacturer, is not guaranteed or endorsed by the publisher.

## Supplementary material

The Supplementary material for this article can be found online at: https://www.frontiersin.org/articles/10.3389/fpsyg.2023.1205913/full#supplementary-material

## Footnotes

^1^Shimamura (2012) states aesthetics has generally been associated in regard to art inducing an emotional response. However, this encompasses more than artworks and in the broader sense refers to an array of objects that can be considered for their beauty. Further, Baumgarten (1750) notes the physical features of an object can elicit a sense of beauty, which comes about as a result of an aesthetic experience.

^2^With regard to the conspicuousness of viewing scenes, research has explored image saliency with regards to how low-level features (e.g., colour, contrast, luminosity, orientation, etc.) predict viewing behavior (see Einhäuser and König, 2003; Itti, 2005; Tatler, 2009; Hayes and Henderson, 2021).

^3^ https://osf.io/ajr48/

^1^These materials are available upon request from the corresponding author.
