# Supplementary material for: Reading in the city: mobile eye-tracking and evaluation of text in an everyday setting
Source: Front Psychol. 2023 Oct 18;14:1205913. doi: 10.3389/fpsyg.2023.1205913 (PMC10622808; doi:10.3389/fpsyg.2023.1205913)
Supplement: Supplementary file 1 [file Data_Sheet_1.docx]

Supplementary Material

Reading in the city: mobile eye-tracking and evaluation of text in an everyday life setting

Kirren Chana*, Jan Mikuni, Alina Schnebel, Helmut Leder

*** Correspondence:** Kirren Chana: kirren.chana@univie.ac.at

| **Supplementary Table 1.** Photographs depicting changes to the eye-tracking testing sites during data collection | |
| --- | --- |
| Study 1: Mariahilferstraße | |
| 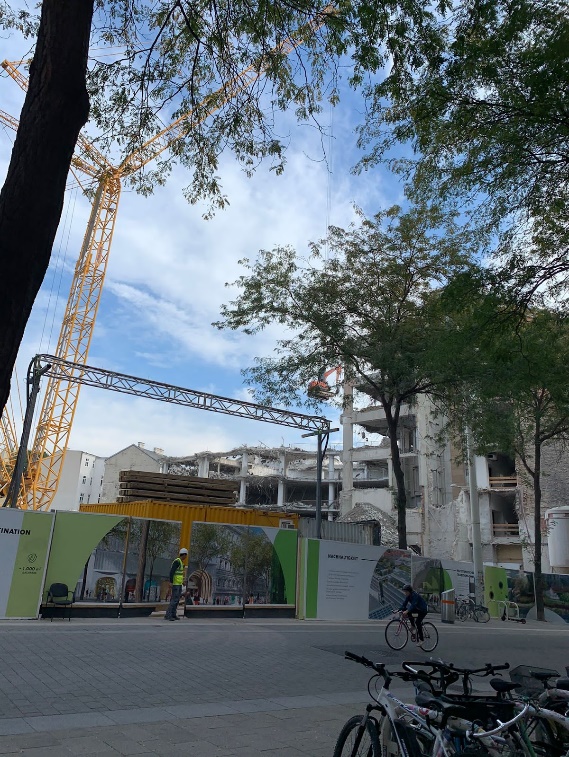 | 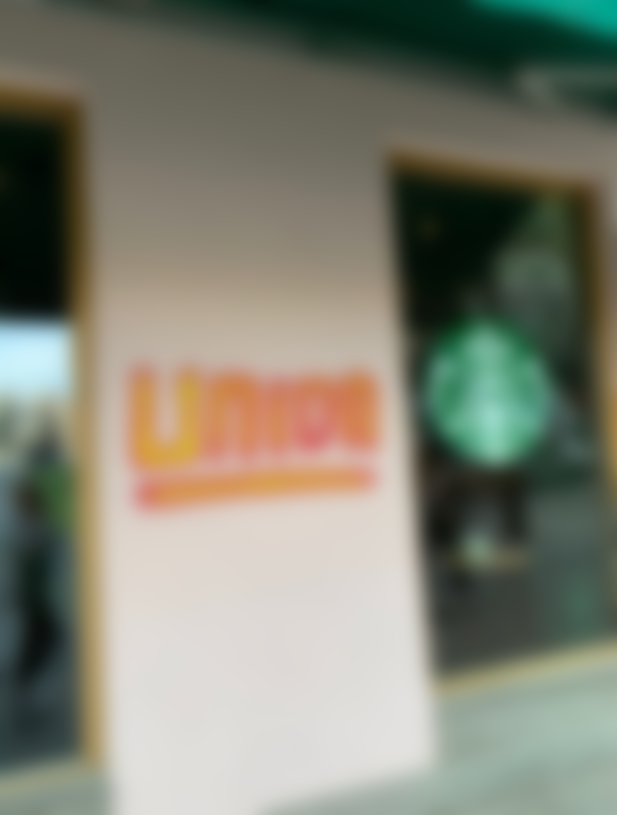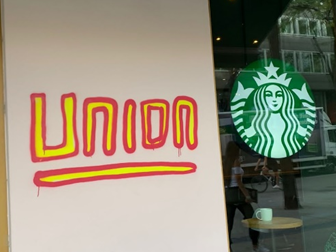 |
| Escalation of work on construction site | Addition (and removal) of graffiti tag |
| 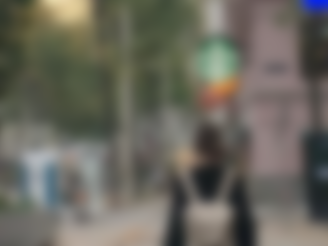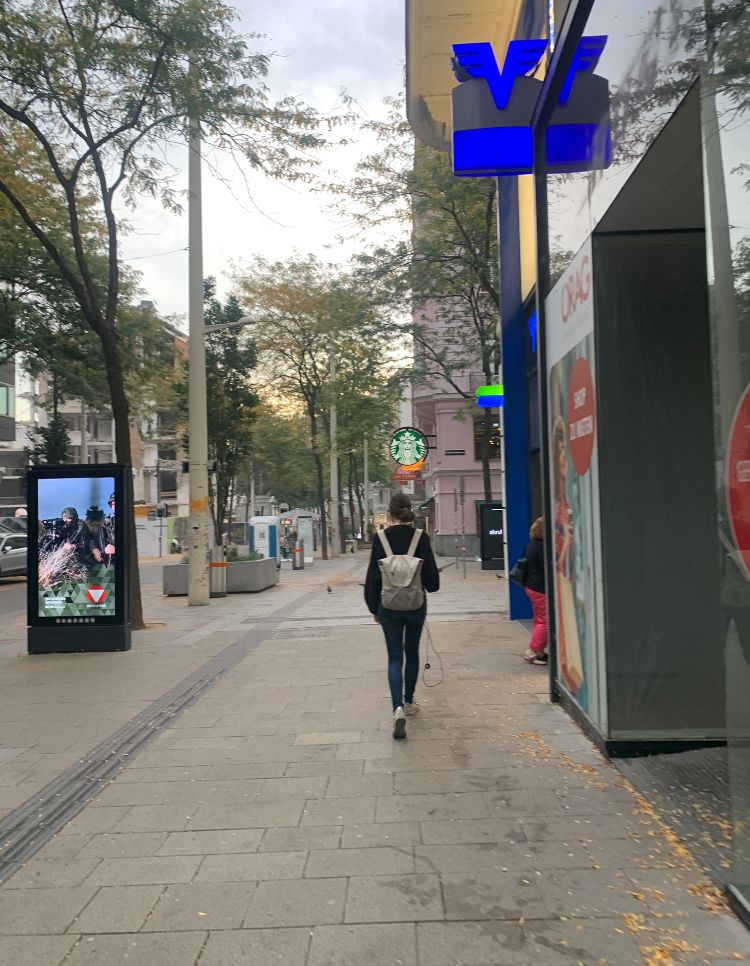 | 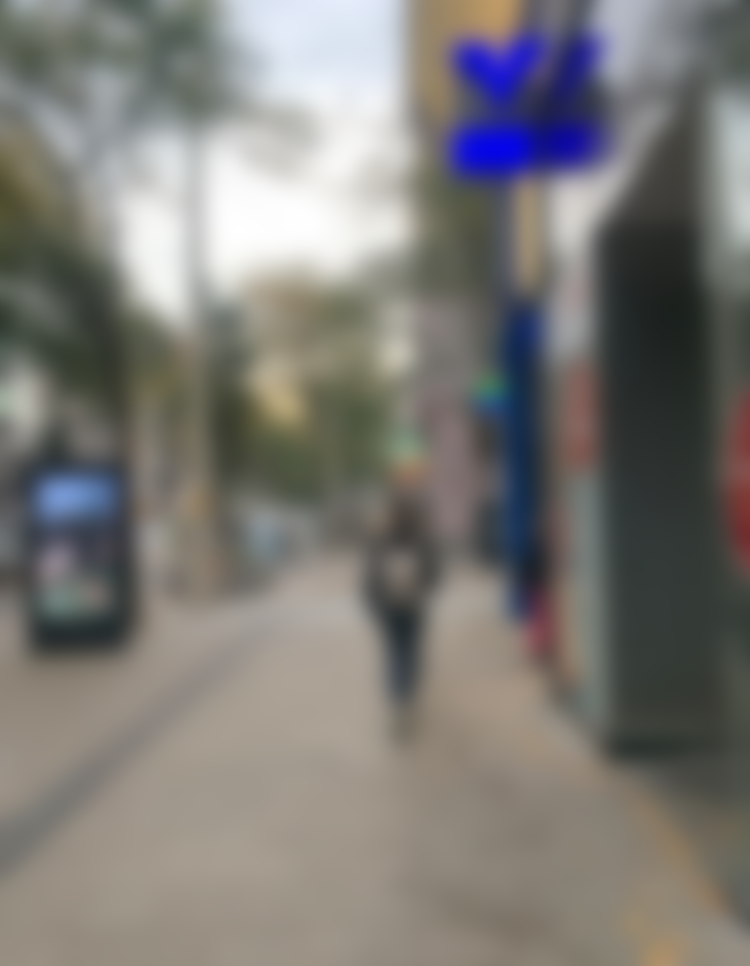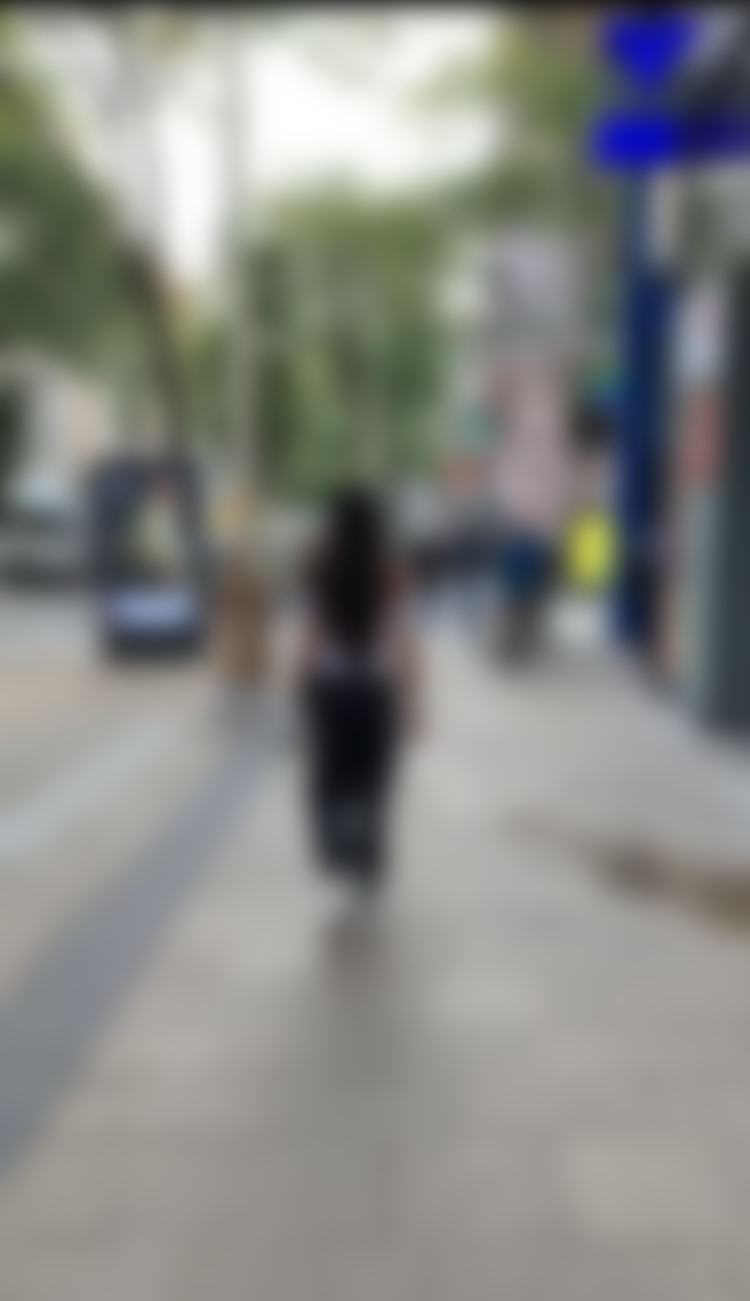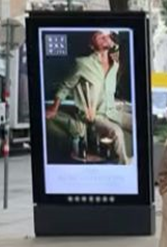 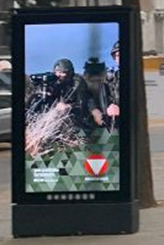 |
| Addition of portable lavatory (bottom left) | Change of advertisements |
| Study 2: Siebensterngasse | |
| 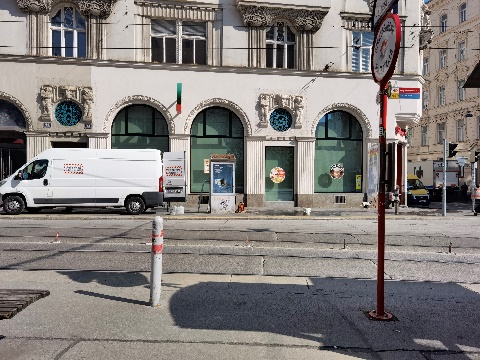  Repair work to drain at starting point of eye-tracking path | 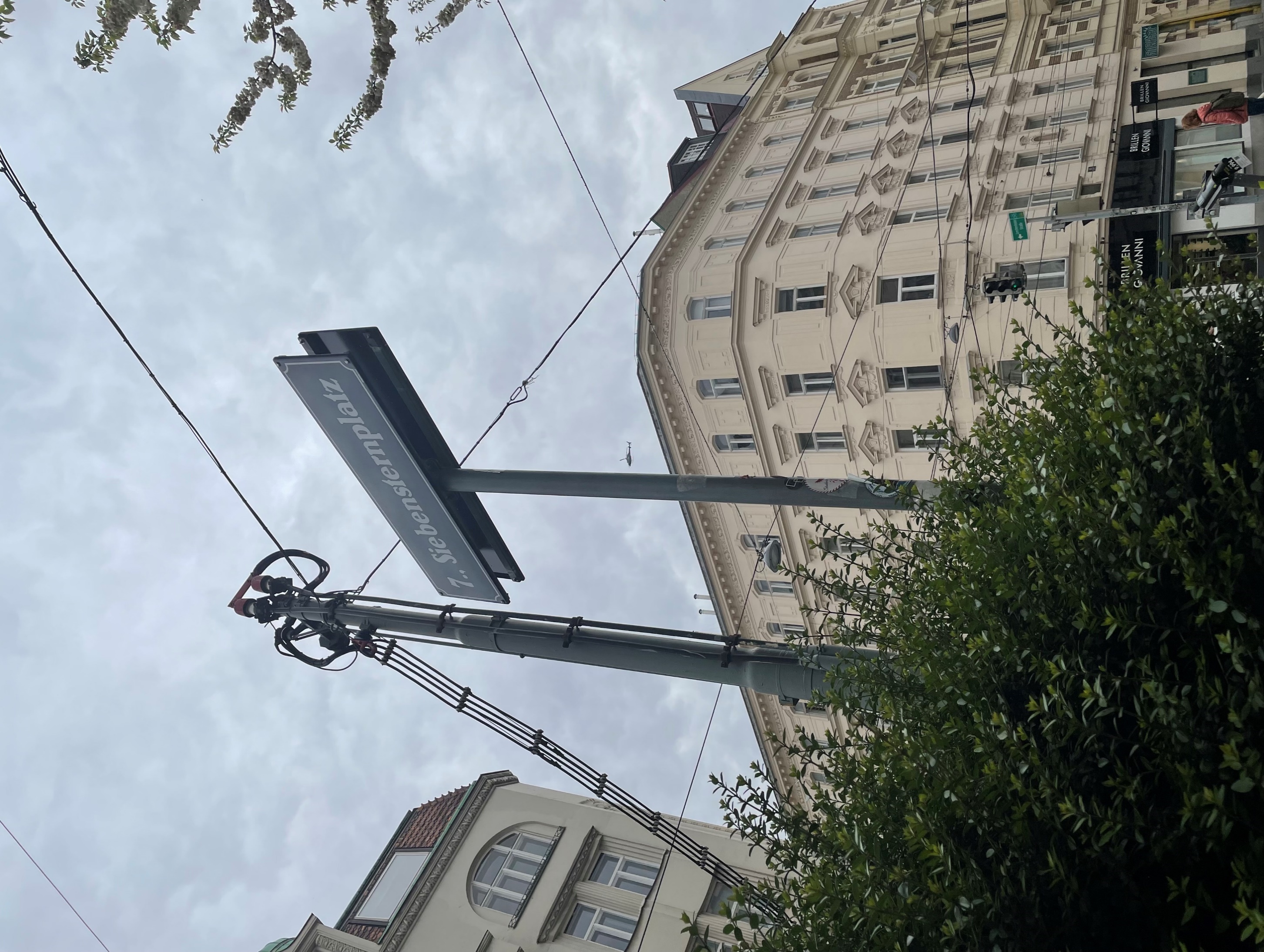  Sound of helicopter circling over surrounding area |
|  | |

**Supplementary Table 2.** Physical characteristics of text sign stimuli (Study 1 and 2)

| Target Sign | Layout | Height (by 1m) | Size | Word length |
| --- | --- | --- | --- | --- |
| ***Study 1*** |  |  |  |  |
| Alisas | flat | 3 | large | 6 |
| Berlitz | protruding | 4 | large | 7 |
| Bijoubrigitte | flat | 2 | large | 13 |
| Bipa | protruding | 3 | small | 4 |
| Brandymelville | flat | 4 | small | 14 |
| Butlers | flat | 3 | large | 7 |
| DerMann | flat | 3 | large | 7 |
| Einbahn | protruding | 3 | small | 7 |
| Interio | protruding | 3 | large | 7 |
| LOccitane | flat | 4 | medium | 9 |
| Misttelefon | flat | 1 | small | 11 |
| Offerl | flat | 2 | small | 6 |
| Phoever | protruding | 4 | large | 7 |
| Pizze | flat | 1 | small | 5 |
| Prokopp | protruding | 3 | large | 7 |
| Snacks | protruding | 2 | small | 6 |
| Starbucks | flat | 3 | large | 9 |
| Sunmiami | protruding | 6 | small | 8 |
| Togo | protruding | 1 | small | 4 |
| Volksbank | flat | 4 | large | 9 |
| ***Study 2*** |  |  |  |  |
| adlerhof | flat | 5 | large | 8 |
| arnolds | protruding | 4 | small | 7 |
| babyyoumakememelt | flat | 3 | large | 17 |
| bealittleanalog | flat | 1 | small | 15 |
| cafenil | flat | 4 | large | 7 |
| cafevoodoo | flat | 3 | large | 10 |
| chicohangematten | flat | 3 | small | 16 |
| glaserei | flat | 4 | large | 8 |
| habibihawara | flat | 4 | large | 12 |
| hotelkugel | flat | 2 | large | 10 |
| inakent | flat | 2 | small | 7 |
| lorenzi | flat | 4 | large | 7 |
| r_s | flat | 4 | small | 3 |
| scharfsinn | flat | 3 | large | 10 |
| schmuckstuck | flat | 3 | large | 12 |
| spar | protruding | 4 | small | 4 |
| tabaktrafik | flat | 4 | large | 11 |
| toms | protruding | 4 | small | 4 |
| ungerade | flat | 4 | large | 8 |
| wellnesscoiffeur | protruding | 3 | small | 16 |

| **Supplementary Table 3.** Total number of fixations according to different fixation criteria | | | |
| --- | --- | --- | --- |
|  | Fixation threshold cut off | | |
|  | All | 100ms | 200ms |
| Study 1 | 8530 | 2553 | 1381 |
| Study 2 | 11001 | 7355 | 5148 |


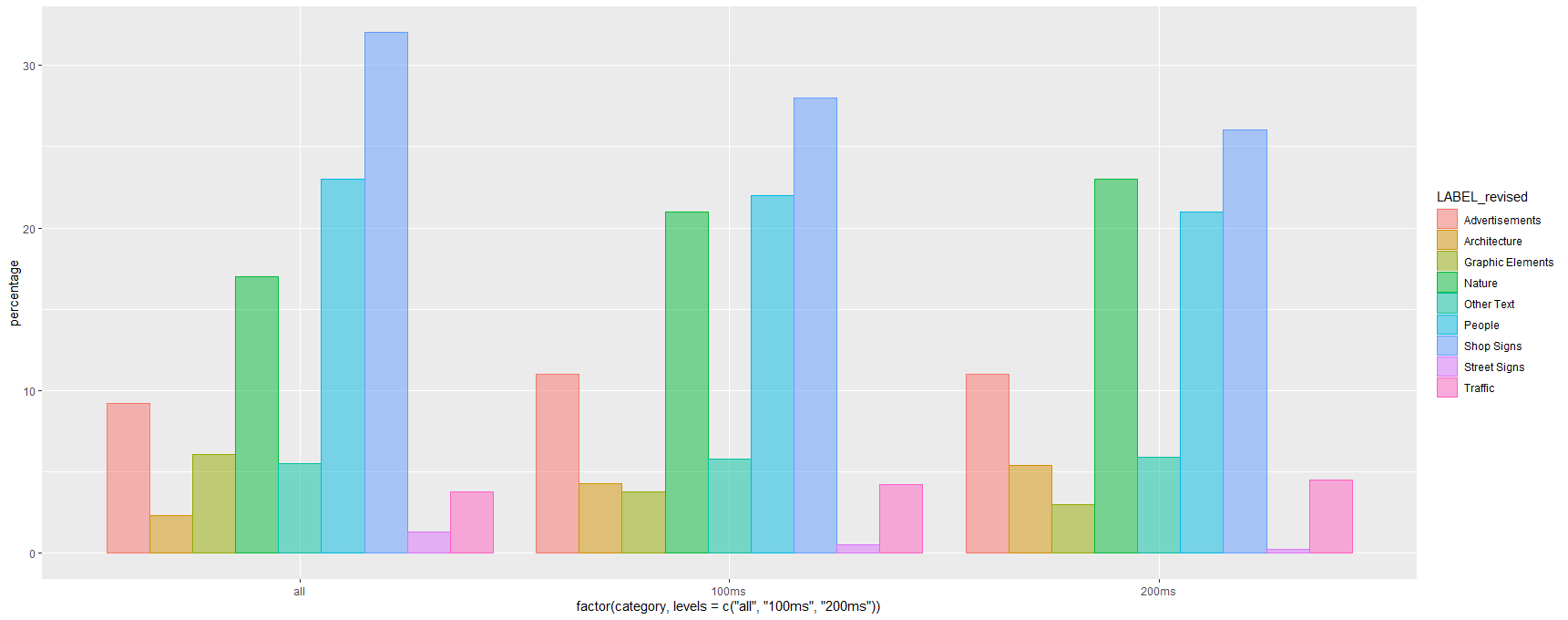


**Supplementary Figure 1.** Study 1: differences in percentage of total numbers of fixations (per category) according to different fixation criteria


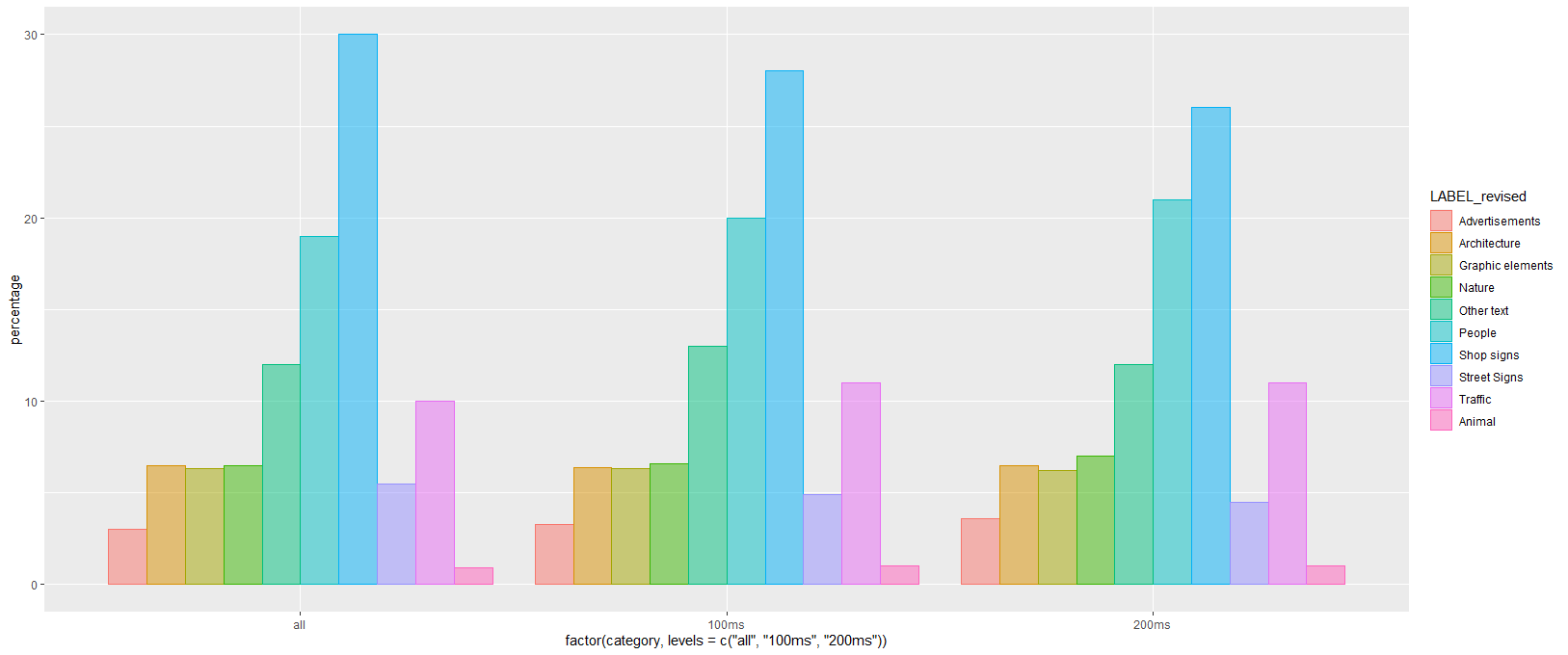


**Supplementary Figure 2.** Study 2: differences in percentage of total numbers of fixations (per category) according to different fixation criteria

| **Supplementary Table 4.** Means and standard deviations for evaluations of text sign stimuli (Study 1, Mariahilferstraße & distractors) | | | | | | | | | | | | | | |
| --- | --- | --- | --- | --- | --- | --- | --- | --- | --- | --- | --- | --- | --- | --- |
| Text Sign | Beauty | | Interest | | Meaningfulness | | Liking | | Attractiveness | | Emotionality | | Familiarity | |
|  | M | SD | M | SD | M | SD | M | SD | M | SD | M | SD | M | SD |
| Alisas | 3.53 | 1.66 | 3.24 | 1.46 | 2 | 1.09 | 2.87 | 1.44 | 3 | 1.61 | 2.03 | 1.03 | 1.97 | 1.73 |
| Berlitz | 2.16 | 0.89 | 1.95 | 1.14 | 1.82 | 1.14 | 2.11 | 1.29 | 2.08 | 1.05 | 1.66 | 1.12 | 3.05 | 2.07 |
| Bijoubrigitte | 3.53 | 1.45 | 3.34 | 1.74 | 3.11 | 1.83 | 3.29 | 1.63 | 3.37 | 1.6 | 3.11 | 1.75 | 6.18 | 1.63 |
| Biogena | 3.16 | 1.52 | 2.97 | 1.42 | 2.05 | 1.47 | 3.18 | 1.35 | 3.08 | 1.46 | 2.37 | 1.46 | 2.55 | 1.81 |
| Bipa | 3.76 | 1.79 | 4.16 | 1.97 | 5.08 | 1.7 | 3.58 | 1.93 | 3.39 | 1.94 | 3.97 | 1.85 | 6.95 | 0.23 |
| Blumen | 3.62 | 1.55 | 3.95 | 1.75 | 4.03 | 1.82 | 3.68 | 1.87 | 3.3 | 1.84 | 3.92 | 1.98 | 3.32 | 2.08 |
| Bohnel | 2.42 | 1.06 | 2.08 | 1.15 | 1.61 | 0.89 | 2.26 | 1.11 | 2.18 | 1.16 | 1.74 | 0.89 | 2.05 | 1.41 |
| Brandymelville | 4.81 | 1.68 | 4.57 | 1.63 | 3.54 | 2.16 | 4.43 | 1.74 | 4.57 | 1.63 | 4.27 | 2 | 5.65 | 2.25 |
| Butlers | 4.46 | 1.82 | 4.43 | 1.72 | 4.08 | 2.13 | 4.57 | 1.72 | 4.43 | 1.71 | 4.03 | 1.91 | 5.95 | 2 |
| Casamexico | 3.58 | 1.41 | 3.29 | 1.63 | 2.61 | 1.65 | 3.37 | 1.58 | 3.32 | 1.58 | 2.55 | 1.7 | 2.68 | 1.85 |
| Christ | 3.18 | 1.31 | 3 | 1.49 | 2.82 | 1.52 | 3.05 | 1.39 | 3.11 | 1.61 | 2.71 | 1.43 | 5 | 2.34 |
| DerMann | 4.03 | 1.48 | 4.47 | 1.52 | 4.76 | 1.6 | 4.24 | 1.68 | 4.05 | 1.82 | 4.11 | 1.87 | 6.66 | 1.02 |
| Dudas_co | 1.81 | 0.94 | 2.11 | 1.29 | 1.62 | 1.06 | 1.81 | 1.08 | 1.51 | 0.8 | 1.84 | 1.32 | 1.43 | 1.12 |
| Einbahn | 3.08 | 1.82 | 4.37 | 1.88 | 5.16 | 1.76 | 3.68 | 1.9 | 3.16 | 1.9 | 3.29 | 2 | 6.89 | 0.39 |
| Gibian | 2.87 | 1.6 | 2.53 | 1.67 | 1.84 | 1.17 | 2.53 | 1.69 | 2.45 | 1.66 | 1.82 | 1.27 | 1.97 | 1.33 |
| H&M | 3.74 | 1.62 | 4 | 1.89 | 4.76 | 1.82 | 3.97 | 1.76 | 3.76 | 1.85 | 4.45 | 1.61 | 6.84 | 0.72 |
| Iamjd | 3.08 | 1.22 | 3.37 | 1.53 | 2.37 | 1.22 | 2.97 | 1.37 | 3.08 | 1.38 | 2.74 | 1.29 | 3.68 | 2.22 |
| Interio | 3 | 1.63 | 3.16 | 1.87 | 3.26 | 2.05 | 3.08 | 1.75 | 2.89 | 1.78 | 2.97 | 1.84 | 5.29 | 2.36 |
| Joseffelber | 2.89 | 1.5 | 2.97 | 1.72 | 2.18 | 1.37 | 2.84 | 1.5 | 2.58 | 1.48 | 2.66 | 1.63 | 2.39 | 1.95 |
| Jugendstil | 4.13 | 1.61 | 4.13 | 1.63 | 2.76 | 1.6 | 3.82 | 1.81 | 3.71 | 1.86 | 3.29 | 1.63 | 2.55 | 1.9 |
| Kunstwerkstatt | 3.55 | 1.16 | 3.76 | 1.4 | 2.58 | 1.2 | 3.53 | 1.27 | 3.39 | 1.35 | 3 | 1.38 | 2.13 | 1.44 |
| LOccitane | 5.42 | 1.24 | 4.79 | 1.58 | 3.97 | 1.81 | 5.21 | 1.56 | 5.32 | 1.47 | 4.18 | 1.84 | 6.26 | 1.22 |
| Mauerer | 2.82 | 1.74 | 3.29 | 2.08 | 1.71 | 0.93 | 2.95 | 1.74 | 2.74 | 1.77 | 2.32 | 1.38 | 1.95 | 1.71 |
| Misttelefon | 2.71 | 1.51 | 3.18 | 1.81 | 3.66 | 2.13 | 3.42 | 1.9 | 2.53 | 1.52 | 3.05 | 1.83 | 5.87 | 1.89 |
| Offerl | 4.66 | 1.6 | 4.39 | 1.91 | 4.11 | 2.18 | 4.61 | 1.85 | 4.47 | 1.93 | 3.68 | 2.09 | 4.53 | 2.5 |
| Phoever | 4.35 | 1.65 | 4.65 | 1.77 | 3.97 | 1.72 | 4.16 | 1.61 | 4.11 | 1.9 | 3.92 | 1.96 | 4.35 | 2.29 |
| Pizze | 3.89 | 1.94 | 4.87 | 1.76 | 4.29 | 2 | 4.42 | 1.91 | 4.03 | 2.01 | 4.21 | 2.08 | 2.87 | 2.03 |
| Prokopp | 1.39 | 0.59 | 1.39 | 0.55 | 1.32 | 0.66 | 1.37 | 0.49 | 1.18 | 0.39 | 1.58 | 0.86 | 1.29 | 0.61 |
| Qipster | 3.21 | 1.56 | 3.82 | 2.18 | 1.79 | 1.19 | 3.03 | 1.82 | 3.08 | 1.82 | 2.68 | 1.69 | 2.82 | 2.19 |
| Salamander | 2.97 | 1.08 | 2.68 | 1.21 | 2.68 | 1.36 | 2.76 | 1.24 | 2.63 | 1.24 | 2.29 | 1.01 | 5.76 | 1.78 |
| Shanta | 3.16 | 1.61 | 3.38 | 1.82 | 1.92 | 1.19 | 2.97 | 1.54 | 3.03 | 1.66 | 2.24 | 1.4 | 1.51 | 0.93 |
| Snacks | 2.63 | 1.38 | 3.32 | 1.68 | 3.05 | 1.52 | 2.89 | 1.56 | 2.71 | 1.8 | 3.03 | 1.79 | 2.21 | 1.58 |
| Starbucks | 3.92 | 1.4 | 3.59 | 1.77 | 3.81 | 1.93 | 3.65 | 1.86 | 3.7 | 1.68 | 3.89 | 1.59 | 6.92 | 0.28 |
| Sunmiami | 2.29 | 1.33 | 2.29 | 1.54 | 1.58 | 1.15 | 1.97 | 1.33 | 2 | 1.36 | 1.87 | 1.34 | 1.13 | 0.34 |
| Tabak | 4.34 | 1.56 | 3.29 | 1.61 | 3.08 | 1.78 | 3.95 | 1.68 | 4.24 | 1.67 | 2.82 | 1.66 | 4.66 | 2.17 |
| Thurzo | 2 | 1.01 | 2.55 | 1.31 | 1.45 | 0.69 | 2 | 0.93 | 1.95 | 0.84 | 2.11 | 1.33 | 1.42 | 0.72 |
| Togo | 3.24 | 1.81 | 3.92 | 2.1 | 3.92 | 1.98 | 3.68 | 1.99 | 3.26 | 1.87 | 3.34 | 1.82 | 4.58 | 2.21 |
| Uhrmacher | 3.74 | 1.37 | 3.74 | 1.73 | 2.89 | 1.47 | 3.66 | 1.56 | 3.39 | 1.46 | 3.05 | 1.66 | 2.71 | 1.87 |
| Volksbank | 3.26 | 1.5 | 3.39 | 1.72 | 3.21 | 2 | 2.92 | 1.62 | 2.95 | 1.56 | 3.18 | 1.87 | 6.61 | 1.05 |
| Waterdrop | 5.11 | 1.16 | 4.16 | 1.26 | 2.61 | 1.57 | 4.37 | 1.57 | 4.71 | 1.33 | 2.97 | 1.6 | 4.53 | 2.08 |
|  | | | | | | | | | | | | | | |
|  | | | | | | | | | | | | | | |

| **Supplementary Table 5.** Means and standard deviations for evaluations of text sign stimuli (Study 2, Siebensterngasse & distractors) | | | | | | |
| --- | --- | --- | --- | --- | --- | --- |
| Text Sign | Beauty | | Meaningfulness | | Familiarity | |
|  | M | SD | M | SD | M | SD |
| adlerhof | 3.04 | 1.40 | 1.35 | 0.75 | 1.58 | 0.99 |
| aloisebeseder | 3.69 | 1.26 | 2.50 | 1.79 | 2.04 | 1.31 |
| antiquitaten | 2.81 | 1.79 | 1.81 | 1.44 | 1.96 | 1.75 |
| arnolds | 2.88 | 1.24 | 2.31 | 1.81 | 1.92 | 1.35 |
| babyyoumakememelt | 3.19 | 1.33 | 2.31 | 1.81 | 2.31 | 1.57 |
| bealittleanalog | 2.28 | 1.46 | 1.76 | 1.45 | 1.56 | 1.23 |
| bootservice | 4.04 | 1.64 | 1.81 | 1.36 | 1.85 | 1.26 |
| cafenil | 3.31 | 1.74 | 1.35 | 0.56 | 1.92 | 1.49 |
| cafevoodoo | 2.65 | 1.47 | 1.73 | 1.22 | 1.42 | 0.90 |
| chicohangematten | 3.42 | 1.88 | 2.08 | 1.49 | 1.92 | 1.47 |
| dorfstube | 2.65 | 1.32 | 1.46 | 0.76 | 1.92 | 1.44 |
| elektrobiker | 3.04 | 1.46 | 1.81 | 1.55 | 1.54 | 0.99 |
| elektroweber | 2.92 | 1.57 | 2.50 | 1.82 | 1.54 | 0.95 |
| felicitas | 2.19 | 1.39 | 1.50 | 1.03 | 1.92 | 1.29 |
| frisurenzimmer | 2.04 | 1.15 | 2.00 | 1.67 | 1.50 | 0.95 |
| glaserei | 3.50 | 1.86 | 2.08 | 1.70 | 2.35 | 1.90 |
| habibihawara | 2.19 | 1.27 | 1.69 | 1.26 | 1.85 | 1.38 |
| hotelkugel | 2.27 | 1.40 | 1.69 | 1.62 | 1.62 | 1.20 |
| inakent | 4.85 | 1.52 | 2.08 | 1.52 | 2.54 | 1.65 |
| kohlenweber | 4.77 | 1.56 | 3.50 | 2.35 | 2.38 | 1.50 |
| kosmetik | 2.81 | 0.90 | 1.77 | 1.31 | 1.54 | 0.86 |
| kosmetiksalon | 3.92 | 1.74 | 3.42 | 2.52 | 2.54 | 1.82 |
| kraftfahrzeuge | 4.50 | 1.98 | 2.65 | 2.13 | 2.69 | 1.76 |
| lecoup | 2.62 | 1.65 | 3.58 | 2.25 | 2.50 | 1.48 |
| lorenzi | 2.50 | 1.10 | 2.96 | 1.99 | 2.15 | 1.35 |
| malbedarf | 2.92 | 1.26 | 1.54 | 1.10 | 2.15 | 1.49 |
| manimaker | 2.88 | 1.97 | 2.19 | 1.58 | 1.85 | 1.32 |
| nagele_strubell | 3.62 | 1.53 | 4.88 | 2.27 | 2.88 | 1.66 |
| proantik | 3.04 | 1.34 | 1.77 | 1.14 | 1.81 | 1.47 |
| r_s | 2.69 | 1.05 | 3.27 | 2.09 | 2.12 | 1.28 |
| scharfsinn | 2.84 | 1.57 | 2.36 | 1.63 | 1.84 | 1.25 |
| schmuckstuck | 3.23 | 1.34 | 1.38 | 0.57 | 1.42 | 0.90 |
| spar | 2.23 | 1.61 | 1.62 | 1.17 | 1.73 | 1.31 |
| tabaktrafik | 4.00 | 1.50 | 2.58 | 1.94 | 2.08 | 1.32 |
| toms | 3.16 | 1.37 | 6.92 | 0.28 | 4.80 | 1.89 |
| tumi | 3.62 | 1.63 | 2.77 | 2.23 | 2.38 | 1.92 |
| ungerade | 2.58 | 1.39 | 2.46 | 2.16 | 1.69 | 1.29 |
| vinonudo | 2.73 | 1.69 | 1.46 | 0.76 | 2.35 | 1.50 |
| wellnesscoiffeur | 1.85 | 0.83 | 1.50 | 0.95 | 1.62 | 0.98 |
| 84karat | 2.77 | 1.50 | 1.50 | 0.99 | 1.62 | 1.06 |

| **Supplementary Table 6.** Distribution of viewing behaviour towards People (Face + Body) in Study 2 | | |
| --- | --- | --- |
|  | Number of Fixations | Percentage (%) |
| Face | 738 | 64 |
| Body | 415 | 36 |
| Total | 1153 | 100 |
| **Note.** Number of fixations with the threshold criterion set at <200ms. | | |
